# Organic-Acid-Modified and PCE-Hybrid Grinding Aids in Slag-Blended Cements: Linking Particle Size Distribution to Hydration and Strength

**DOI:** 10.3390/polym18212674

**Published:** 2026-10-28

**Authors:** Veysel Kobya, Yahya Kaya, Ali Mardani

**Affiliations:** 1 Department of Civil Engineering, Faculty of Engineering, Bursa Uludag University, Bursa 16059, Turkey; 2 Construction Chemistry, Institute for Advanced PCE Studies, Technical University of Munich, 80333 Garching, Germany; 3 Materials Technologies and 3D Concrete Printing R&D Laboratory, Bursa Uludag University, Bursa 16059, Turkey

**Keywords:** granulated blast-furnace slag, grinding aid, organic acid modification, polycarboxylate ether, hydration, compressive strength

## Abstract

Reducing clinker content while maintaining the performance of slag-blended cement requires grinding-aid strategies that improve grinding efficiency and support subsequent hydration. In this study, commercial triisopropanolamine (TIPA), diethanolisopropanolamine (DEIPA), and diethylene glycol (DEG), their organic-acid-modified derivatives, and their hybrids with a polycarboxylate ether (PCE)-based grinding aid were evaluated at 0.05% in cement systems containing two granulated blast-furnace slags (BFSs) with different physical and chemical characteristics. Particle-size distribution, X-ray diffraction, thermogravimetric analysis, scanning electron microscopy, zeta potential, PCE adsorption, and compressive strength tests were used to investigate the relationships between grinding-aid chemistry, particle characteristics, hydration, and mechanical performance. Similar Blaine fineness values did not necessarily yield comparable particle-size distributions or hydration responses, indicating that Blaine fineness alone is insufficient to assess the effectiveness of grinding aids in BFS-blended cement. Organic-acid modification was generally more effective than PCE hybridization, depending on the original grinding-aid chemistry. M-TIPA promoted the strongest hydration development and the highest 28- and 56-day strengths at all BFS replacement levels. At 30% replacement, M-TIPA mixtures with BFS 1 and BFS 2 reached 28-day strengths of 45.82 and 46.55 MPa and 56-day strengths of 52.69 and 54.13 MPa, respectively, exceeding the clinker-only control. At 45% replacement, modified alkanolamines reduced, but did not eliminate, strength loss, whereas clinker dilution dominated at 60%. Overall, grinding-aid selection should consider particle-size distribution, hydration, BFS characteristics, and replacement level, rather than relying on Blaine fineness alone, and should also account for additive chemistry and the potential influence of polymer-containing formulations on the interfacial properties of ground cement particles.

## 1. Introduction

Reducing carbon emissions from cement production has become a key priority in the construction industry’s transition toward low-carbon technologies. The environmental burden of Portland cement is primarily associated with direct CO_2_ emissions from limestone calcination and the substantial energy required for clinker production. According to the International Energy Agency (IEA), achieving net-zero emissions requires reducing the global clinker-to-cement ratio from approximately 0.71 in 2022 to 0.65 by 2030 [1]. Within the constraints of existing cement production infrastructure, partial replacement of clinker with supplementary cementitious materials (SCMs) represents one of the most practical and effective strategies for achieving this target [2,3,4]. Nevertheless, lowering the clinker content should not be assessed solely from an environmental perspective. Excessive clinker substitution may reduce early-age strength, delay formwork removal, or require higher binder dosages to achieve the desired mechanical performance, thereby offsetting part of the anticipated environmental benefits. Consequently, the successful development of low-clinker binders requires minimizing clinker consumption while maintaining adequate hydration kinetics and mechanical performance [5,6].

Ground granulated blast-furnace slag (GGBFS) is one of the most extensively used latent hydraulic SCMs in Portland cement systems due to its high amorphous phase content and its CaO–SiO_2_–Al_2_O_3_–MgO-based composition. The incorporation of GGBFS reduces clinker consumption [7]. It promotes the formation of C-(A)-S-H gel, AFm phases, and Mg-Al hydrotalcite-like hydrates during the later stages of hydration [8]. These hydration products contribute to pore refinement, reduced permeability, and enhanced durability against aggressive environmental conditions [9,10]. Unlike Portland clinker, however, GGBFS does not hydrate rapidly upon contact with water. Instead, its dissolution is governed by the alkalinity and ionic composition of the pore solution, the nature of the reaction products formed on the slag surface, and the availability of ions released during clinker hydration [11]. Consequently, at replacement levels of approximately 40–60%, the combined effects of clinker dilution and the inherently slower hydration kinetics of slag often result in a pronounced reduction in early-age strength [12,13,14].

The reactivity of GGBFS depends on several factors beyond its replacement level, including amorphous phase structure, CaO/SiO_2_ ratio, Al_2_O_3_ and MgO contents, thermal history, specific surface area, and particle size distribution, which collectively govern slag dissolution [15,16,17,18,19]. Although higher basicity generally promotes glass dissolution, higher SiO_2_ contents may retard early dissolution by forming a more polymerized glass network. Al_2_O_3_ and MgO also influence the formation of C-(A)-S-H, AFm, and hydrotalcite-like phases. However, chemical composition alone cannot fully predict slag reactivity, as cooling history, glass structure, and particle size can lead to substantially different hydration behaviors [17,18,19].

Particle size reduction accelerates early hydration by increasing the reactive surface area of slag and clinker [20]. However, Blaine fineness alone does not adequately describe the grinding state, as cements with similar values may exhibit different particle-size distributions, particularly in the ultrafine and coarse fractions. A high D90 indicates a greater proportion of coarse particles, which have limited reactive surface area during early hydration [21,22,23,24]. Therefore, particle fractions, D_50_, D_90_, and distribution characteristics should be evaluated collectively. This is particularly important for GGBFS-containing binders, where coarser slag particles may further restrict early hydration and intensify the dilution effect at high replacement levels.

Increasing fineness may also promote particle re-agglomeration due to the high surface energy generated during grinding, reducing grinding efficiency [25,26,27,28]. Grinding aids (GAs) mitigate these effects by adsorbing onto newly formed surfaces, reducing inter-particle attraction, and improving powder flow. Thus, grinding performance, energy consumption, and particle size distribution depend strongly on GA chemistry and dosage [29,30,31]. Previous studies by the research group have similarly shown that amine-, glycol-, and PCE-based GAs can produce distinct particle-size distributions despite comparable Blaine fineness, leading to differences in fresh-state properties and strength development [24,32,33]. The role of GAs extends beyond grinding. During hydration, alkanolamines interact with Ca^2+^-, Al-, and Fe-bearing species, influencing clinker dissolution, hydrate nucleation, and the aluminate–sulfate balance [34]. TIPA generally enhances medium- and late-age strength, whereas DEIPA preferentially accelerates aluminate and ferrite hydration but may delay C_3_S hydration depending on dosage and cement composition [35,36]. These effects are governed by adsorption behavior, sulfate availability, solution chemistry, and hydrate layer characteristics rather than ion complexation alone [23,37,38,39]. In contrast, DEG primarily acts through physical mechanisms, reducing agglomeration and improving powder flow, with its influence on hydration arising mainly from the resulting particle-size distribution [40].

Recent research has focused on improving GA performance through the organic acid modification of alkanolamines and glycols. Esterification alters molecular polarity, adsorption characteristics, and particle interactions, thereby affecting grinding performance [41,42]. Although ester-modified additives can improve cement fineness and particle size distribution, their effectiveness depends on the organic acid used, the degree of modification, and the dosage [43,44]. Moreover, enhanced grinding efficiency does not necessarily translate into faster hydration or higher strength, as residual organic species on cement surfaces may either promote or hinder subsequent hydration reactions [45].

The second approach involves combining conventional grinding aids with PCE-based additives [46]. In addition to electrostatic interactions, adsorbed PCE molecules reduce fine particle re-agglomeration through steric hindrance provided by their side chains [47,48,49]. However, their effectiveness depends strongly on molecular parameters, including backbone length, anionic charge density, side-chain length, anionic/non-ionic ratio, pH, and dosage [50,51]. Appropriate PCE structures at low dosages can increase the fine fraction, whereas excessive adsorption or unsuitable molecular characteristics may negatively affect grinding efficiency, hydration, and admixture compatibility [51,52,53,54,55]. Therefore, additive selection cannot be based solely on grinding performance, as hydration and strength development may vary with changes in pH, dosage, and mineral admixture composition [56].

In GGBFS-containing systems, the effects of grinding aids are governed by the combined contributions of clinker hydration, slag dissolution, and particle size distribution. Although alkanolamines can accelerate clinker hydration and increase alkalinity, slag reactivity is controlled by additional factors, including Ca^2+^ availability, sulfate concentration, dissolution of Al-bearing species, and the formation of C-(A)-S-H layers on slag surfaces [9,12]. Accordingly, changes in portlandite formation should not be directly interpreted as an indicator of the degree of slag reaction [13]. Recent studies have shown that alkanolamine dosage has a narrow optimum range in GGBFS systems. Low TEA dosages can enhance strength development by improving particle-size distribution and slag reactivity, whereas excessive dosages may retard silicate hydration and disrupt the aluminate–sulfate balance [57,58]. These findings demonstrate that grinding aid performance should be evaluated not only by additive chemistry and dosage but also by the slag’s mineralogical characteristics and particle-size distribution. Key studies and their relevance to the present work are summarized in Table 1.

Previous studies have investigated commercial alkanolamine- and glycol-based grinding aids, organic acid-modified additives, and PCE-based grinding aids individually. However, most investigations have focused on a single BFS type, a limited range of additives, or an isolated evaluation of grinding performance and strength development. Consequently, the relationships among grinding performance, particle-size distribution, hydration development, and mechanical performance have not been systematically compared within a common experimental framework. In particular, an increase in conventional fineness indicators, such as Blaine fineness, does not necessarily indicate a comparable refinement of the particle-size distribution or improved subsequent hydration and strength development. Systematic comparisons of different grinding-aid strategies within a single experimental framework, using BFS with distinct chemical compositions and finenesses, remain scarce. This knowledge gap is particularly relevant for BFS-containing cement systems because the effects of grinding-aid modification may depend on the physical and chemical characteristics of the BFS. Therefore, the improvements in fineness and hydration observed in conventional cement systems cannot be assumed to be maintained when less basic or coarser BFSs are incorporated.

Accordingly, the primary objective of this study is to systematically establish the relationships among grinding-aid chemistry, modification strategy, grinding performance, particle-size distribution, and hydration and strength development in BFS-blended cement. The primary objective of this study is to identify grinding aid formulations that mitigate early-age hydration and strength losses while maintaining the clinker-reduction benefits associated with high BFS replacement levels. For this purpose, commercial TIPA, DEIPA, and DEG; their organic acid-modified derivatives (M-TIPA, M-DEIPA, and M-DEG) prepared using hexanoic and propanoic acids; and PCE-blended additives (P-TIPA, P-DEIPA, and P-DEG) produced by combining commercial additives with a PCE-based grinding aid at a 1:1 mass ratio were systematically evaluated. The influence of these additives was assessed not only by Blaine fineness but also by particle-size distribution parameters, including fine fractions, D50, D90, and the Rosin–Rammler distribution coefficient.

Furthermore, two BFS types with different chemical compositions, basicity, and particle fineness were incorporated at a 45% replacement level to evaluate hydration development using XRD, TGA, and SEM analyses at 24 h, 3 days, and 28 days. Zeta-potential and PCE adsorption measurements were additionally performed to provide indirect information on the interfacial properties of the ground cements. Compressive strength development was investigated in systems with and without chemical admixtures at BFS replacement levels of 0%, 30%, 45%, and 60% over curing periods of 1, 3, 7, 28, and 56 days. Through this integrated approach, it is examined whether improvements in grinding performance achieved through additive modification are reflected in particle-size distribution and subsequently translated into enhanced hydration and mechanical performance, whether these relationships remain valid for BFSs with different characteristics and replacement levels, and how portlandite evolution relates to strength development under different additive–BFS combinations. By linking grinding-aid modification and the interfacial behavior of PCE-containing systems to the performance of slag-blended cement, this study provides a comparative basis for developing more effective grinding-aid formulations for clinker-reduced cement production.

## 2. Materials and Methods

### 2.1. Materials

#### 2.1.1. Grinding Aids

Three groups of GAs were used in this study: commercial additives, organic acid-modified additives, and hybrid systems obtained by combining commercial additives with polycarboxylate ether (PCE)-based GAs. The commercial GA group consisted of TIPA, DEIPA, and DEG.

Molecular characterization of the synthesized PCE was performed using gel permeation chromatography (GPC) (Malvern–OmniSEC, United Kingdom (England) to determine its molecular weight distribution and polydispersity index (PDI), following the procedure described in the literature [50]. The PCE solution had a solid content of 30.12%, a pH of 3.54, a viscosity of 172 cP, and a density of 1.078 g/mL. GPC analysis yielded a weight-average molecular weight (Mw) of 37,000 Da and a number-average molecular weight (Mn) of 17,600 Da, corresponding to a PDI of 2.1. The main-chain and side-chain molecular weights were 4000 and 2400 Da.

The solid content was determined by drying the sample at 105 °C to constant mass. The pH was measured at 25 °C using a calibrated pH meter (BIOBASE, China), and the density was determined using a density meter. The molecular weight parameters were obtained by GPC.

The second group comprised modified GAs selected based on findings from previous studies [42,51]. TIPA and DEIPA were esterified with hexanoic acid, whereas DEG was modified with propanoic acid. The resulting products were denoted as M-TIPA, M-DEIPA, and M-DEG, respectively. For the modification process, commercial GAs were reacted with the corresponding carboxylic acid in the presence of sulfuric acid as a catalyst in a 250 mL two-neck glass reactor at 85 °C for 3 h. The water generated during esterification was continuously removed under vacuum to promote the reaction. The organic acid-modified grinding aids investigated in this study were synthesized using the procedure previously developed by the authors. Detailed chemical characterization of these modified grinding aids, including FTIR, ^13^C NMR, and GC–MS analyses, was reported in our previous study [53]. These complementary analyses confirmed the formation of the corresponding ester-modified structures. Therefore, the present study does not repeat the complete structural characterization of the synthesized products, but focuses on their performance in cement grinding and cementitious systems.

The third group consisted of PCE-based GAs whose grinding performance and molecular characteristics had been previously determined [40]. Hybrid GAs were prepared by physically blending the PCE-based GA with commercial TIPA, DEIPA, and DEG at a 1:1 mass ratio, resulting in P-TIPA, P-DEIPA, and P-DEG, respectively. All GAs, including the hybrid systems, were used at a dosage of 0.05% by mass relative to the total mass of clinker and gypsum during grinding. Accordingly, in the hybrid systems, the individual dosages of PCE and the corresponding commercial GA were 0.025% by mass relative to clinker and gypsum. The manufacturer-reported properties of the commercial chemicals are summarized in Table 2a, while the molecular characteristics of the PCE-based GA are presented in Table 2b.

#### 2.1.2. Clinker, Gypsum, and Blast Furnace Slags

A cement consisting of 96% clinker and 4% gypsum by mass was used for cement production. Grinding was performed to obtain the target fineness for a control cement produced without GA and nine GA-containing cement formulations.

Two types of BFS with different chemical compositions and particle-size characteristics were used in this study and designated as BFS1 and BFS2. For hydration and microstructural investigations, BFS1 and BFS2 were incorporated as 45% mass replacement of the GA-ground cements.

The chemical compositions and selected physical properties of the clinker, gypsum, and BFS samples, as provided by the manufacturer, are presented in Table 3.

#### 2.1.3. Aggregate

Washed natural river sand with a particle size range of 0–4 mm was used as the fine aggregate in the mortar mixtures. The saturated surface-dry (SSD) specific gravity and water absorption of the aggregate were determined in accordance with TS EN 1097-6 [60] and measured at 2.40 and 1.20%, respectively.

### 2.2. Methods

Clinker grinding was performed using a Bond-type laboratory ball mill (Mikroanaliz, Ankara) with a motor power of 1.5 kW and a maximum capacity of 5 kg. The control cement was produced by grinding a mixture of 96% clinker and 4% gypsum by mass without any additives. For the GA-containing cement series, the corresponding GA was added to the clinker–gypsum mixture before grinding at a dosage of 0.05% by mass relative to the total mass of clinker and gypsum.

The specific surface area of the produced cements was determined using an automatic Blaine analyzer (Mikroanaliz, Ankara), while particle size distributions were measured using a Malvern Zetasizer Nano ZSP (Malvern, Eng) based on laser diffraction analysis. Prior to each laser diffraction measurement, the sample was ultrasonically dispersed for 5 min to minimize particle agglomeration and obtain a more representative particle-size distribution. The same pretreatment and dispersion procedure was applied to all samples to ensure consistent and comparable measurement conditions. The particle-size distributions were characterized using the D10, D50, and D90 values, along with the Rosin–Rammler distribution parameter. The equation describing the cumulative particle size distribution according to this model is given in Equation (1):

(1) $$R\;=\;\exp\left[-{\left(\frac{x}{x^{1}}\right)}^{n}\right]$$

Here, R(x) represents the cumulative mass fraction of particles coarser than size x; X^l^ represents the characteristic particle size corresponding to 63.2% passing; and n represents the distribution slope parameter. A higher n value indicates a narrower and more uniform particle size distribution, whereas a lower n value represents a broader distribution.

To indirectly assess GA adsorption during grinding, the adsorption of the PCE-type water-reducing admixture onto cement particles was quantified using total organic carbon (TOC) analysis, following the procedure described by Feng et al. [53]. In addition, the zeta potential (ZP) of the cement samples was measured using a Zetasizer ZS90 instrument at a constant temperature of 23 °C. The applied voltage was adjusted within the range of 50–100 V. The measurements were performed following standard procedures reported in the literature.

To investigate the time-dependent evolution of hydration products and microstructural development, cement pastes were prepared at a constant water-to-binder ratio (w/b) of 0.35 [34,54]. For BFS-containing mixtures, the binder was considered as the total mass of cement and BFS. After casting, the samples were kept in a curing cabinet at 20 ± 2 °C and 95 ± 2% relative humidity for 24 h. The specimens were then demolded and continuously cured in water at room temperature until the designated testing ages of 3 and 28 days.

At each testing age, the specimens were fractured into approximately 1 cm^3^ pieces and immersed in isopropanol for 48 h to stop further hydration. After solvent treatment, samples for XRD and TGA analyses were ground to particle sizes below 90 µm and dried at 40 °C for 48 h. For SEM analysis, fractured but ungrounded specimens were examined to preserve the original morphology of the hydration products and microstructural features.

XRD and TGA were used to analyze the prepared powder samples. XRD was used to identify the evolution of crystalline phases associated with clinker and hydration products, whereas TGA was evaluated based on mass losses occurring within specific temperature intervals. The portlandite reflection intensity was used only as a relative indicator of comparison between mixtures; Rietveld refinement was not performed. SEM observations were performed to characterize the microstructures of the paste, the morphology of hydration products, and the interactions between BFS particles and the surrounding hydration phases.

Mortar mixtures for compressive strength testing were prepared in accordance with ASTM C109 [61]. Washed natural river sand with a particle size range of 0–4 mm was used as fine aggregate instead of standard sand. All mortar mixtures were prepared at a constant w/b ratio of 0.45 and a binder-to-sand ratio of 1:3. The flow diameter of each mortar mixture was measured in accordance with ASTM C1437 [62] and maintained within the range of 220 ± 20 mm. The target flow was achieved by adjusting the PCE dosage for each mixture, while the water and binder contents were kept constant. Thus, the effective w/b ratio remained constant among all mixtures, ensuring comparable conditions for compressive strength evaluation. All mixtures were therefore prepared using the PCE dosage required to achieve the target flow (220 ± 20 mm) at the fixed w/b and binder-to-sand ratios. The specimens were subsequently cured under standard water-curing conditions until the specified testing ages. The compressive strength of mortar mixtures was determined in accordance with the ASTM C109 procedure. For each mixture, the compressive strength was determined from three specimens, and the average value was reported as the representative strength.

## 3. Results and Discussions

### 3.1. Particle Size Distributions

**Figure 1:**
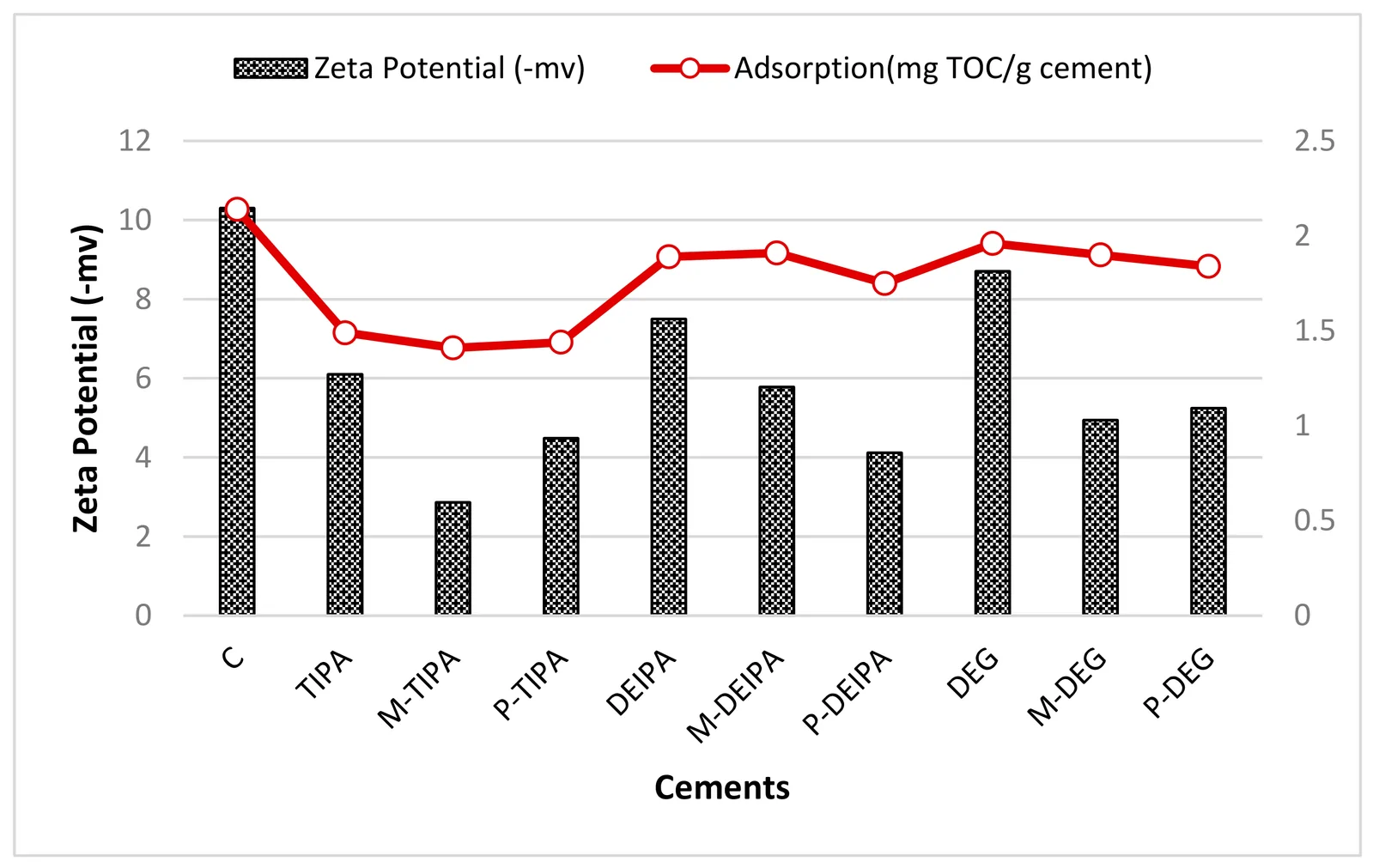
Zeta potential and PCE adsorption of the cements ground without and with GAs.

The Blaine specific surface areas and particle size distribution parameters of the control cement (without GA), GA-containing cements produced using a dosage of 0.05% by mass relative to the total clinker and gypsum content, and the BFS samples used in this study are presented in Table 4. In addition, to indirectly investigate the potential adsorption of GA onto clinker particles during grinding and its relationship with PSD, Figure 1 presents the zeta potential and PCE adsorption results for the ground cements.

Although GAs caused only a limited increase in Blaine specific surface area, they significantly modified the particle size distribution of the cements. The Blaine values increased from 3810 cm^2^/g for the control cement to 3840–3940 cm^2^/g for GA-containing cements, corresponding to an increase of only 0.8–3.4%. However, variations in D50, D90, and particle size fractions demonstrate that GAs influence the distribution of ground particles rather than merely increasing total surface area. Therefore, cement fineness cannot be adequately evaluated using Blaine values alone. By adsorbing onto newly formed clinker surfaces, GAs can reduce particle re-agglomeration and adhesion to the grinding media, thereby improving grinding efficiency and particle size reduction [24,32].

The control cement exhibited 68.28% of particles below 32 µm and 19.29% above 45 µm. With the TIPA addition, the <32 µm fraction increased to 76.66%, while the >45 µm fraction decreased to 11.14%. In parallel, D50 and D90 decreased from 17.3 to 14.9 µm and from 69.9 to 49.2 µm, respectively, corresponding to reductions of 13.9% and 29.6%. The increase in the Rosin–Rammler parameter from 0.804 to 0.931 confirms the formation of a narrower particle size distribution. These results indicate that TIPA effectively limits fine particle re-agglomeration and promotes the reduction in coarse clinker particles. This is consistent with Figure 1, which shows that TIPA achieved the largest reduction in PCE adsorption (30%) among the commercial GAs, indicating high surface coverage.

M-TIPA increased the <32 µm fraction to 75.92% and reduced the >45 µm fraction to 10.99%; however, its effect on D50 and D90 (16.5 and 55.2 µm, respectively) was lower than that of commercial TIPA. This suggests that hexanoic acid modification alters particle distribution but does not confer the same efficiency in reducing the coarse-particle fraction. Previous studies have shown that organic acid modification affects additive polarity and adsorption behavior depending on the original molecule and acid type [42,63]. Figure 1 shows that M-TIPA and TIPA exhibited similar PCE adsorption amounts (1.41 and 1.49 mg/g, respectively), but different ζ-potentials (−2.86 and −6.10 mV). This difference may indicate changes in the interfacial properties of the cement particles and the structure of the adsorbed layer, although these measurements cannot directly confirm the latter.

For P-TIPA, D50 and D90 were 16.7 and 56.1 µm, respectively, while the fraction of particles larger than 45 µm decreased to 13.91%. Although P-TIPA produced a finer particle size distribution than the control cement, its effectiveness in increasing the fine fraction was lower than that of TIPA and M-TIPA. This behavior may be partly attributed to the reduced TIPA content in the hybrid system. The potential contribution of competitive adsorption between TIPA and PCE remains uncertain, as it could not be directly verified experimentally. Therefore, the grinding performance of P-TIPA may reflect the combined effects of PCE-induced steric stabilization and TIPA-related surface interactions within the hybrid system.

Within the DEIPA group, commercial DEIPA exhibited the highest Blaine fineness (3940 cm^2^/g); however, its D90 value (64.6 µm) decreased by only 7.6% compared with the control cement. This confirms that a higher Blaine value does not necessarily indicate a lower coarse particle fraction. Although DEIPA increased the fine fraction, it was less effective than TIPA in reducing the coarse particle tail. Similarly, M-DEIPA showed only a limited effect on coarse-particle reduction, with a D90 of 68.2 µm. However, the increase in the Rosin–Rammler parameter (n = 0.964) indicates a more uniform particle-size distribution than in the control cement.

P-DEIPA provided the greatest improvement in fineness within the DEIPA group, reducing D50 and D90 to 15.1 and 53.9 µm, respectively, corresponding to decreases of 12.7% and 22.9% compared with the control cement. Although its ultrafine fraction was slightly lower than that of commercial DEIPA, the lower D50 and D90 values indicate that PCE hybridization effectively modified the medium- and coarse-particle regions. This agrees with previous studies showing that appropriately designed PCE-based GAs can reduce agglomeration and improve particle size distribution through steric stabilization [40,54].

For DEG, the D50 and D90 values were 16.5 and 56.3 µm, respectively. Unlike TIPA and DEIPA, DEG did not increase the ultrafine fraction but reduced the >45 µm fraction to 16.74%, suggesting that its primary contribution is the reduction in coarse particles and improvement of powder flow rather than the formation of ultrafine particles. This behavior is consistent with the absence of amine functionality in DEG, indicating that its grinding mechanism is mainly associated with reducing inter-particle attraction, mill adhesion, and powder flow resistance.

M-DEG exhibited the most favorable particle size distribution among DEG-based additives, with D50 and D90 values of 15.2 and 54.7 µm and the highest n value (0.998) among all tested cements. Compared with the control cement, D50 and D90 decreased by 12.1% and 21.7%, respectively. These results suggest that propanoic acid modification improves DEG performance by narrowing the particle size distribution and reducing the coarse fraction. However, a high n value does not necessarily indicate a higher content of ultrafine particles.

In contrast, P-DEG increased the <32 µm fraction to 73.69%, yielding an n value of 0.978; however, the D90 increased to 77.7 µm. The increased coarse-particle fraction indicates that the PCE-DEG combination was less effective at controlling the coarse region of the distribution. This behavior suggests that PCE compatibility depends on the molecular structure of the commercial GA, and differences in surface adsorption, additive distribution within the mill, or interactions between PCE and DEG may influence the final grinding performance.

The results demonstrate that GA performance cannot be evaluated using a single fineness parameter. While Blaine’s specific surface area reflects overall surface-related characteristics, D50, D90, particle-size fractions, and the Rosin–Rammler parameter provide complementary information across different regions of the particle-size distribution. For example, although DEIPA exhibited the highest Blaine value, its D90 performance was inferior to that of TIPA, M-TIPA, P-TIPA, P-DEIPA, DEG, and M-DEG. Likewise, the high n value of P-DEG, despite its high D90, indicates that distribution uniformity alone does not characterize the coarse particle fraction. Therefore, grinding performance should be assessed by considering Blaine fineness, particle-size fractions, D50, D90, and distribution parameters collectively.

Particle size distribution also determines the initial physical conditions for hydration. Although finer particles generally promote faster dissolution due to their larger reactive surface area, improved hydration and strength cannot be predicted solely from lower D50 and D90 values. Residual GA molecules on cement surfaces may influence the dissolution of clinker phases and the formation of hydrates in various ways. González-Panicello and Palacios [36] showed that TIPA and DEIPA can produce different hydration and microstructural outcomes even at comparable fineness levels. Therefore, the hydration effects of the particle size variations presented in Table 4 were further evaluated using XRD, TGA, SEM, and compressive strength analyses.

Compared with ground cements, BFS1 and BFS2 exhibited coarser particle size distributions. BFS1 showed a Blaine value of 3200 cm^2^/g, D50 of 27.2 µm, and D90 of 112 µm, whereas BFS2 exhibited values of 3500 cm^2^/g, 22.2 µm, and 87.5 µm, respectively. The lower D50 and D90 values and reduced >45 µm fraction of BFS2 (20.75% compared with 23.76% for BFS1) indicate a finer particle distribution. Since BFS dissolution is slower than clinker hydration, fineness plays an important role in early-age reactivity by controlling the available reaction surface and dissolution of Si- and Al-containing species. Previous studies have demonstrated that increased slag fineness accelerates early reaction and microstructural development [18,19,64]. Accordingly, BFS2 is expected to exhibit higher early-age reactivity based solely on its physical characteristics.

However, slag reactivity cannot be explained solely by particle size. The higher CaO/SiO_2_ ratio and nominally higher basicity of BFS2 may contribute to a more favorable dissolution environment; however, this effect cannot be isolated from the substantially finer particle-size distribution and the differences in Al_2_O_3_ and MgO contents between the two slags. Therefore, both physical and chemical differences between BFS1 and BFS2 were considered when interpreting subsequent hydration and strength results.

Overall, Table 4 shows that TIPA was the most effective additive for reducing the coarse particle fraction, whereas P-DEIPA and M-DEG yielded a more balanced particle-size distribution. Although M-TIPA generated a high fine fraction and reduced coarse particles, it did not exceed commercial TIPA in terms of D50 and D90. Similarly, P-DEG increased the fine fraction but was less effective at controlling coarse particles than other hybrid systems. These findings indicate that the effectiveness of organic acid modification and PCE hybridization depends strongly on the chemical structure of the base GA, and identical modification strategies do not necessarily produce comparable outcomes for TIPA, DEIPA, and DEG.

All GAs made the zeta potential of the ground cements less negative (−2.86 to −8.70 mV vs. −10.30 mV for the control) and reduced the amount of PCE taken up by the cements (1.41–1.96 vs. 2.14 mg TOC/g). Both trends are consistent with GA molecules being present on the particle surfaces and occupying sites that would otherwise be available for PCE adsorption; PCE uptake is therefore used here as an indirect indicator of GA surface coverage. The effect was largest for the TIPA family (30–34% lower PCE uptake) and smaller for DEIPA and DEG (8–18%), in line with the larger reduction in the >45 µm fraction obtained with TIPA-based GAs (Table 4; ρ = 0.78, *p* = 0.013 for the GA-containing cements). Organic-acid modification did not change PCE uptake relative to the parent GA (differences ≤ 0.08 mg/g) but made ζ less negative in all three families (by 1.7–3.8 mV), suggesting similar surface coverage but a different structure of the adsorbed layer, which agrees with reports that esterification alters polarity and adsorption behavior [42,63]. The PCE hybrids showed ζ values 1.6–3.5 mV less negative than the corresponding single GAs, consistent with PCE adsorption and displacement of the shear plane by its side chains [47,48,49]. It should be noted that the TOC measurements quantify the uptake of a PCE water reducer by the ground cements and therefore provide only indirect information on GA surface coverage; they do not resolve the adsorption of the individual components of the hybrid GAs or any competition between them. Accordingly, the hydration effects of the hybrid GAs discussed in Section 3.3, Section 3.4, Section 3.5 and Section 3.6 are not attributed specifically to PCE adsorption or to competitive adsorption.

### 3.2. Mineralogical and Chemical Characterization of Blast Furnace Slags

**Figure 2:**
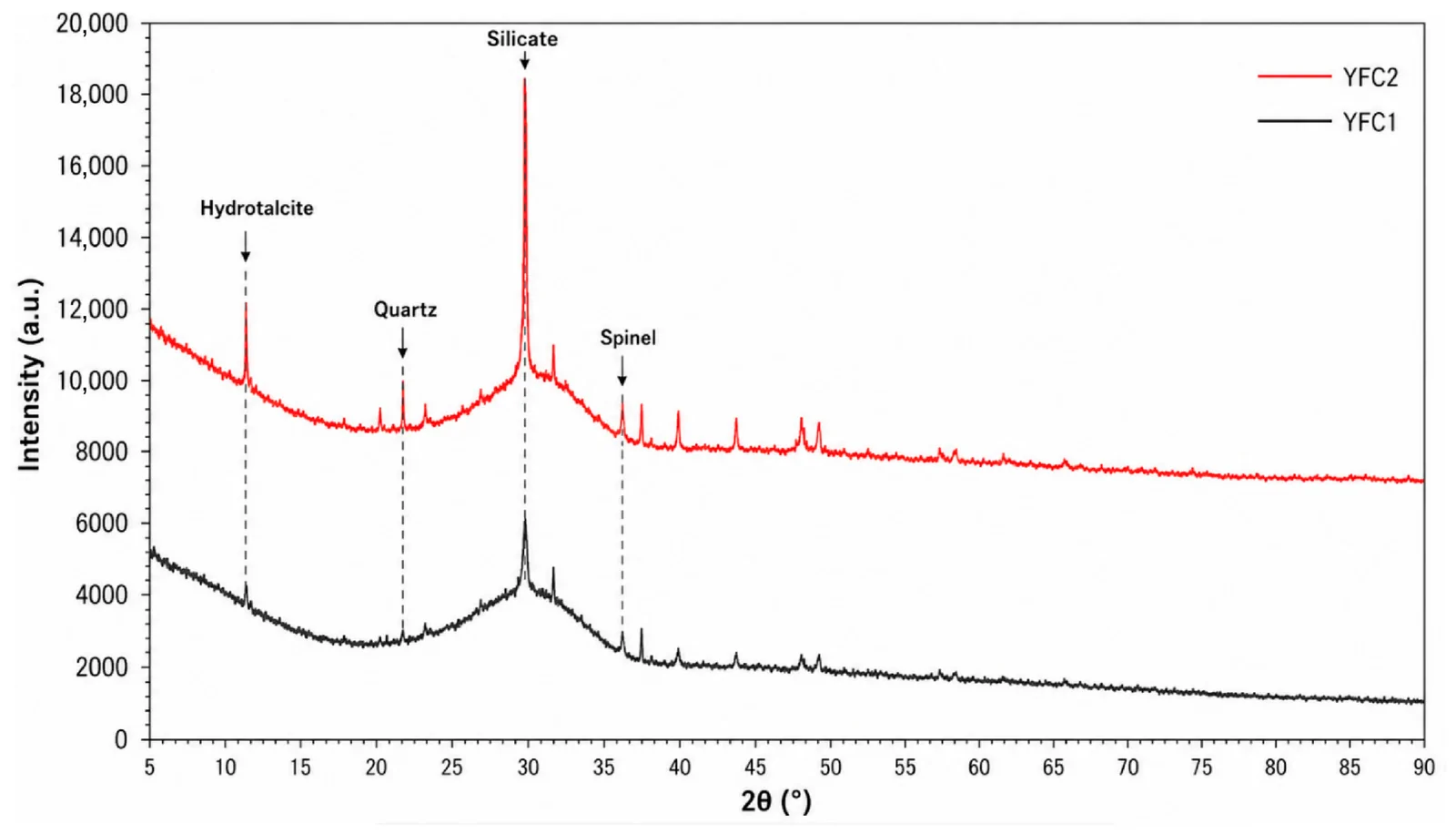
XRD patterns of BFS1 and BFS2.

The chemical compositions of BFS1 and BFS2, as determined by XRF analysis, are presented in Table 3, and their XRD patterns are shown in Figure 2.

The broad scattering band observed between approximately 20–35° 2θ in the XRD patterns of both BFS samples confirms the predominance of a disordered Ca-Mg-Al-Si-based glassy phase, which is responsible for the latent hydraulic behavior of granulated blast-furnace slag. However, this feature alone does not quantify the glass content or predict reactivity, as slag hydration is also governed by the glass structure, chemical composition, particle fineness, and pore-solution chemistry [7,9,12].

Low-intensity crystalline reflections indicate the presence of minor crystalline phases in both BFS samples. The reflection near 20.8–21° 2θ is associated with quartz, whereas peaks around 29–30° and 36–37° 2θ may correspond to secondary silicate, carbonate, or spinel phases formed during cooling. The weak reflection near 11° 2θ suggests the possible presence of hydrotalcite-like phases formed during limited moisture exposure of the stored slag. However, this single reflection is insufficient to determine the amount of this phase or its influence on subsequent hydration behavior [65].

The chemical compositions of BFS1 and BFS2 represent typical CaO–SiO_2_–Al_2_O_3_–MgO-based slag systems [7], but notable differences were observed. BFS1 contained higher SiO_2_ (38.91%), Al_2_O_3_ (14.24%), and MgO (7.68%) contents, whereas BFS2 exhibited higher CaO content (40.52% compared with 32.09% for BFS1). Consequently, BFS1 has a more silica-, alumina-, and magnesia-rich composition, whereas BFS2 has a higher calcium content, which may influence dissolution behavior and the formation of hydration products.

The CaO/SiO_2_ ratios were calculated as 0.82 for BFS1 and 1.18 for BFS2, indicating a higher nominal basicity for BFS2. Although higher basicity can facilitate glass dissolution by modifying the silicate network, the CaO/SiO_2_ ratio alone cannot predict the reactivity of the present slags. The dissolution behavior of BFS is also influenced by the degree of polymerization and structural connectivity of the glassy phase, the Al_2_O_3_ and MgO contents, cooling history, and particle-size distribution. Therefore, the higher CaO/SiO_2_ ratio of BFS2 is considered as one of several potentially contributing factors rather than as a direct indicator of its higher reactivity [7,9,66].

The ((CaO + MgO)/SiO_2_) ratios were 1.02 and 1.32 for BFS1 and BFS2, respectively, while the total CaO + MgO + SiO_2_ contents were 78.68% and 80.00%. Therefore, both slags satisfy the requirements of EN 15167-1, including a ((CaO + MgO)/SiO_2_) ratio above 1.0 and a total CaO + MgO + SiO_2_ content exceeding two-thirds of the mass. However, the higher nominal basicity of BFS2 should be considered alongside its finer particle-size distribution and different Al_2_O_3_ and MgO contents when assessing its potential reactivity.

The higher Al_2_O_3_ content of BFS1 may promote Al incorporation into C-(A)-S-H structures and the formation of AFm-type phases, whereas its higher MgO content may favor the formation of Mg-Al-containing hydrotalcite-like phases [9,65,67]. Nevertheless, the formation of these phases depends not only on total oxide content but also on the distribution of oxides within the glassy phase, the degree of slag reaction, and the pore-solution chemistry.

The chemical differences between BFS1 and BFS2 are further supported by their particle size characteristics. BFS2 exhibited a higher Blaine fineness (3500 cm^2^/g) compared with BFS1 (3200 cm^2^/g), together with lower D50 and D90 values (22.2 and 87.5 µm for BFS2; 27.2 and 112 µm for BFS1). Thus, BFS2 exhibits a finer particle distribution and a lower fraction of coarse particles, thereby providing a larger reactive surface area during early hydration. Previous studies have demonstrated that increased slag fineness enhances early-age slag reaction and strength development [7,12,18].

BFS2’s finer particle size distribution, higher CaO content, and higher CaO/SiO_2_ ratio provide more favorable initial conditions for early-age reaction compared to BFS1. BFS1’s higher Al_2_O_3_ and MgO content, on the other hand, may alter the composition of the C-(A)-S-H, AFm, and hydrotalcite-like phases that form at later ages [65,67,68]. The impact of these differences on hydration development and strength results was evaluated using subsequent XRD, TGA, SEM, and compressive strength measurements.

### 3.3. XRD Results of Mixtures

The values in Table 5 and Table 6 represent the portlandite peak intensities obtained from the XRD patterns. The peak intensities were used not as an indication of the absolute amount of portlandite, but to compare portlandite development under the same sample preparation and measurement conditions.

#### 3.3.1. BFS1 Paste Mixtures

The peak portlandite strengths at 24 h, 3 days, and 28 days for pastes containing and not containing BFS1 are presented in Table 5.

Portlandite peak intensity increased with curing time for all investigated mixtures, indicating the progressive development of crystalline portlandite during clinker hydration. This evolution was particularly associated with the hydration of calcium silicate phases, mainly C_3_S. The control paste showed a progressive increase in the characteristic portlandite reflection from 24 h to 28 days, as also observed for the grinding-aid-containing systems (Table 5).

Among the BFS-free mixtures, M-TIPA exhibited the most pronounced development of the characteristic portlandite reflection at all curing ages, followed by the other TIPA-based systems. Commercial TIPA also promoted the development of crystalline portlandite compared with the control paste, although the effect was less pronounced than that observed for M-TIPA. The more pronounced portlandite development observed for M-TIPA, despite its relatively larger D50 and D90 values compared with TIPA, suggests that its effect cannot be attributed solely to particle-size refinement. The enhanced hydration observed for M-TIPA could be related to a change in the additive’s interaction with clinker surfaces and ions in the pore solution following hexanoic acid modification, an interpretation consistent with earlier reports [42,44].

A similar qualitative trend was observed for the DEIPA-based additives. M-DEIPA showed more pronounced development of the portlandite reflection than commercial DEIPA and P-DEIPA. Since this behavior was not accompanied by a corresponding reduction in D90 relative to commercial DEIPA, the observed difference cannot be attributed exclusively to particle-size refinement. The enhanced hydration associated with M-DEIPA may therefore also be related to changes in chemical and surface interactions introduced by organic acid modification. The interaction of alkanolamines with Ca-, Al-, and Fe-containing species can influence clinker dissolution and subsequent precipitation processes, depending on the additive structure, dosage, and cement composition.

For the DEG-containing mixtures, commercial DEG exhibited a portlandite evolution broadly comparable to that of the control paste, consistent with the absence of an amine functionality in DEG and its distinct interaction mechanism relative to TIPA and DEIPA. In contrast, M-DEG showed more pronounced development of the portlandite reflection, suggesting enhanced clinker hydration. This behavior may be associated with the combined effects of particle-size modification and changes in surface interactions resulting from propanoic acid modification. P-DEG, in contrast, did not show a comparable enhancement, indicating that PCE hybridization did not provide an additional beneficial effect on the development of crystalline portlandite in the DEG-based system.

The P-TIPA, P-DEIPA, and P-DEG mixtures generally exhibited less pronounced portlandite development than the corresponding systems containing the commercial additives alone. This behavior may be partly related to the constant total grinding-aid dosage used in the hybrid systems, which resulted in a lower amount of the conventional TIPA, DEIPA, or DEG components than in their corresponding single-component applications. PCE present on the particle surfaces could, in principle, also influence dissolution and hydrate precipitation; however, because the adsorption of the hybrid components was not measured directly, this possible contribution cannot be confirmed or separated from the dosage effect with the present data. The differences among the PCE-based hybrid systems further suggest that the effect of PCE incorporation depends on the chemical nature of the original grinding-aid component.

The incorporation of BFS1 generally reduced the development of the portlandite reflection compared with the corresponding BFS-free mixtures (Table 5). This behavior is expected because replacing 45% of the clinker with BFS reduces the amount of clinker available for hydration, thereby reducing the potential formation of portlandite during clinker hydration. In addition, the latent-hydraulic reaction of BFS may consume part of the portlandite generated during clinker hydration. Therefore, the lower portlandite peak intensities observed in the BFS1-containing systems may result from the combined effects of clinker dilution and subsequent portlandite consumption during BFS reaction.

The relatively pronounced development of the portlandite reflection at early ages in some BFS-containing mixtures also indicates that the reduction cannot necessarily be interpreted as a simple dilution effect. At a constant water-to-binder ratio, clinker replacement increases the amount of water available per unit mass of the remaining clinker, while the presence of BFS may influence the hydration environment and provide additional surfaces for the precipitation of hydration products. Nevertheless, these effects cannot be quantitatively distinguished from the XRD peak intensities alone.

At later ages, the lower portlandite peak intensities of the BFS1-containing mixtures are consistent with the combined effects of clinker dilution and the ongoing reaction of BFS. During BFS hydration, additional calcium aluminosilicate hydrate phases, AFm phases, and Mg-Al-containing hydrotalcite-like phases may develop, and portlandite may participate in these reactions [9,65,68]. Thus, the lower intensity of the portlandite reflection in BFS-containing systems does not necessarily indicate a proportional reduction in clinker hydration.

The qualitative trends observed for the BFS1-containing mixtures were generally consistent with those of the corresponding BFS-free systems. In particular, M-TIPA-BFS1-45 exhibited the most pronounced development of the portlandite reflection among the TIPA-based BFS1 mixtures, while M-DEIPA-BFS1-45 also showed more pronounced portlandite development than the corresponding commercial and PCE-based systems. This indicates that the effects of organic-acid modification of the grinding aids were maintained with a 45% BFS1 replacement.

It should be noted that the XRD peak intensities presented in Table 5 are used only to assess the qualitative evolution of crystalline portlandite. Because diffraction intensity can be influenced by factors such as preferred orientation, crystallinity, and sample-related effects, these values are not interpreted as direct measures of CH content. The quantitative differences in CH formation among the investigated systems are therefore evaluated using the TGA results, which provide a more appropriate basis for determining CH content from the characteristic dehydroxylation region.

#### 3.3.2. BFS2 Paste Mixtures

The peak Portlandite strengths of pastes with and without BFS2 at 24 h, 3 days, and 28 days are presented in Table 6.

As observed for BFS1, the BFS2 substitution reduced the relative intensity of the portlandite reflections across the investigated additive groups. However, considering that XRD peak intensity can be affected by factors such as preferred orientation, crystallinity, and instrumental conditions, these differences were not interpreted as direct quantitative changes in portlandite content. Instead, the XRD results were used primarily to identify the evolution of crystalline hydration phases and to provide supporting evidence for the hydration behavior of the investigated systems. The quantitative evaluation of portlandite formation and consumption was performed using TGA results, based on mass loss in the 400–550 °C range.

In the BFS2 control mixture (without additional admixtures), the portlandite reflection became progressively more pronounced from 24 h to 28 days, indicating the continued formation of portlandite during clinker hydration. A similar increase in the relative intensity of the portlandite reflection with hydration age was generally observed in the GA-containing mixtures. The differences between the BFS1- and BFS2-containing systems, particularly at later ages, indicate that BFS incorporation influenced the evolution of the hydration products. However, these differences in peak intensity were not used to quantify the extent of portlandite formation or consumption.

The differences observed between the BFS1 and BFS2 systems may be attributable to the distinct physical and chemical characteristics of the two slags. The finer particle-size distribution of BFS2 provides a greater available surface area for interaction with the alkaline pore solution, while its higher CaO/SiO_2_ ratio and basicity may favor the dissolution and reaction of its glassy phase. Consequently, the reaction of BFS2 may contribute more substantially to the formation of secondary hydration products, such as C-(A)-S-H and Al-containing hydrates, particularly at later ages. Previous studies have shown that BFS reactivity is governed by the combined effects of slag fineness, chemical composition, and the ionic composition of the pore solution [9,12]. Nevertheless, the contribution of these mechanisms to portlandite formation and consumption is more appropriately evaluated from the TGA results rather than from XRD peak intensity alone.

Among the BFS2-containing mixtures, M-TIPA-BFS2-45 exhibited a pronounced portlandite reflection across all investigated hydration ages. Although the XRD patterns qualitatively indicate that the hydration-promoting effect associated with M-TIPA was maintained in the presence of BFS2, the relative peak intensity cannot be directly used to quantify the extent of clinker hydration or portlandite consumption. The later-age evolution of the BFS-containing systems may instead reflect the combined effects of clinker hydration, slag reaction, and the formation of additional hydration products. These effects are evaluated quantitatively using the TGA results.

M-DEIPA also exhibited more pronounced portlandite reflections than commercial DEIPA in both BFS-free and BFS2-containing mixtures. A similar qualitative trend was observed for M-DEG compared with commercial DEG. The persistence of these differences in the presence of BFS2 suggests that the modification of the grinding aids affected the subsequent hydration behavior of the resulting cement systems. However, the XRD results alone do not permit quantification of the magnitude of this effect in terms of portlandite content. The observed differences are therefore interpreted together with the TGA results and other characterization techniques [42].

The portlandite reflections of the P-TIPA-BFS2-45 and P-DEG-BFS2-45 mixtures were less pronounced than those of their corresponding mixtures containing commercial additives, whereas P-DEIPA-BFS2-45 showed a similar portlandite reflection to DEIPA-BFS2-45 at 28 days. These observations suggest that PCE hybridization may influence the development of crystalline hydration phases; however, the extent of this influence cannot be reliably quantified from the relative peak intensity. The effect of PCE incorporation on hydration behavior is therefore further assessed using the quantitative TGA results and complementary characterization techniques.

Considering the XRD results obtained for both BFS1 and BFS2 systems, the incorporation of 45% BFS clearly altered the development of the crystalline hydration phases compared with the BFS-free systems. The changes observed with increasing hydration age are consistent with the increasing contribution of slag reaction to the overall hydration process. In particular, the differences between BFS1 and BFS2 may be associated with their different particle-size distributions and chemical compositions, which influence slag dissolution and subsequent hydrate formation. The finer particle-size distribution and higher basicity of BFS2 may promote its reaction with the alkaline pore solution, consistent with previous findings on the effects of slag fineness and composition on BFS reactivity [9,12]. However, the extent of portlandite formation and consumption associated with these processes is discussed primarily based on TGA measurements rather than on XRD peak intensities.

Among the modified additives, M-TIPA generally produced the most pronounced portlandite reflections across the investigated binder compositions, while M-DEIPA and M-DEG also generally exhibited more pronounced reflections than their corresponding commercial additives. These XRD observations provide qualitative support for the differences in hydration behavior among the grinding-aid formulations. Nevertheless, the observed changes in portlandite peak intensity should not be interpreted as direct evidence of changes in portlandite content because of the inherent limitations associated with intensity-based XRD comparisons. Therefore, quantitative differences in portlandite formation and consumption are evaluated using TGA data, while XRD results are used to support the identification and evolution of the crystalline phases.

Overall, the XRD results demonstrate that both BFS incorporation and grinding-aid modification influenced the evolution of the crystalline hydration phases. The differences between BFS1 and BFS2 are consistent with the effects of slag fineness and chemical composition on clinker–slag hydration. In contrast, the differences among the commercial, modified, and PCE-hybridized grinding aids indicate that the modification strategy can influence subsequent hydration behavior. However, the XRD peak intensities were not used to quantify portlandite content in the revised analysis. Instead, the phase evolution observed by XRD was interpreted together with TGA, SEM, and compressive strength results, with TGA providing the primary quantitative assessment of portlandite formation and consumption. This combined approach provides a more reliable basis for discussing the hydration mechanisms of the investigated BFS-containing cement systems.

### 3.4. TGA Results of Mixtures

When evaluating TGA results, the mass loss in the 50–200 °C range is primarily due to the dehydration of C-(A)-S-H, ettringite, AFm, and hydrotalcite-like hydrates, while the loss in the 200–390 °C range is attributed to the decomposition of hydrate phases containing more strongly bound water; and the loss in the 400–550 °C range is primarily associated with the dehydroxylation of portlandite. Since multiple hydrate phases lose water simultaneously in the low-temperature range, the mass loss at 50–200 °C is evaluated not as the direct amount of C-(A)-S-H, but as the total contribution of the hydrates decomposing in this range [9,65,68].

#### 3.4.1. BFS1 Paste Mixtures

Table 7 presents the TGA results at 24 h, 3 days, and 28 days for pastes containing 45% by mass BFS1 as a substitute, produced using control cement and cements containing 0.05% GA.

In mixtures containing BFS1, the incorporation of alkanolamine-based additives increased the mass loss associated with hydration products compared with the additive-free C-BFS1-45 mixture at all hydration ages. At 24 h, the mass loss in the 50–200 °C range increased from 1.260% for C-BFS1-45 to 2.466%, 2.707%, and 2.727% for the TIPA-, M-TIPA-, and P-TIPA-containing mixtures, respectively. It should be noted that the mass loss within this temperature interval cannot be assigned exclusively to a specific hydration phase. In BFS-containing systems, overlapping contributions from C-(A)-S-H, AFm-type phases, other Al-bearing hydrates, and water associated with Mg-Al hydrotalcite-like phases may occur within or near this temperature range. Therefore, the 50–200 °C mass loss is considered here as an overall indicator of thermally labile and bound water rather than a direct quantitative measure of the amount of a particular hydration product. These results suggest that TIPA-based additives promoted hydrate development at early ages despite the high BFS1 replacement level. Furthermore, the CH content of the M-TIPA-BFS1-45 mixture (15.851%) exceeded that of the TIPA-BFS1-45 and P-TIPA-BFS1-45 mixtures, providing complementary evidence of enhanced clinker hydration in the M-TIPA system during the early hydration period.

The differences among the TIPA-based mixtures became more pronounced as curing time increased. The mass loss of the M-TIPA-BFS1-45 mixture in the 50–200 °C range increased to 4.006% at 3 days and 4.456% at 28 days. At 28 days, this mixture also exhibited the highest CH and non-evaporable water (NEW) contents among all BFS1-containing mixtures, reaching 37.862% and 19.438%, respectively, compared with 34.915% and 17.684% for the mixture containing commercial TIPA. Because the NEW value is derived from thermogravimetric mass loss and the contributions of different hydration phases cannot be completely separated using the present temperature intervals, these values should be interpreted as comparative indicators rather than absolute measures of chemically bound water. The enhanced performance of M-TIPA may be attributed to hexanoic acid modification, which alters the adsorption characteristics of TIPA on clinker surfaces and influences ion interactions during hydration, thereby promoting greater hydrate formation. Previous studies have likewise demonstrated that organic acid modification improves grinding efficiency, refines particle size distribution, and enhances early-age hydration [42,44,63].

Although the P-TIPA-BFS1-45 mixture exhibited a higher 28-day mass loss in the 50–200 °C range (4.284%) than the mixture containing commercial TIPA, its CH and NEW contents remained lower, at 29.476% and 14.982%, respectively. This discrepancy suggests that the increased low-temperature mass loss was not solely attributable to greater portlandite formation but also reflected differences in the types and distributions of hydration products. The lower CH and NEW contents of P-TIPA-BFS1-45 are most directly explained by the halved TIPA content of the hybrid formulation (0.025% instead of 0.05%). Literature reports that adsorbed PCE can modify dissolution, nucleation, and hydrate precipitation [46,47,53] suggest a possible additional contribution; however, since neither the adsorption of the PCE component nor its competition with TIPA for surface sites was directly measured, this mechanism remains a hypothesis rather than a demonstrated cause. Therefore, the higher low-temperature mass loss observed for P-TIPA should not be interpreted as evidence of a higher overall degree of hydration.

DEIPA-based additives also enhanced hydration compared with the additive-free C-BFS1-45 mixture. The DEIPA-BFS1-45 mixture exhibited mass losses of 2.634%, 3.899%, and 3.147% in the 50–200 °C range at 24 h, 3 days, and 28 days, respectively. Although the M-DEIPA-BFS1-45 mixture showed a slightly lower early-age mass loss than the commercial DEIPA mixture, it exhibited higher CH and NEW contents at both 3 and 28 days. At 28 days, the CH and NEW contents of M-DEIPA-BFS1-45 reached 30.214% and 15.106%, respectively, compared with 28.905% and 14.216% for DEIPA-BFS1-45. These findings indicate that organic acid modification had a greater influence on portlandite formation and chemically bound water than on the total amount of low-temperature hydration products within the DEIPA system.

A similar trend was observed for the P-DEIPA-BFS1-45 mixture. Although its 28-day mass loss in the 50–200 °C range (3.238%) exceeded those of both the DEIPA- and M-DEIPA-containing mixtures, its CH and NEW contents remained lower than those of M-DEIPA-BFS1-45. As observed for the TIPA-based systems, this behavior suggests that PCE hybridization modifies not only the quantity but also the composition and thermal stability of the hydration products. The simultaneous adsorption of alkanolamine and PCE molecules onto clinker surfaces likely altered ion transport and phase precipitation during the initial stages of hydration compared with the use of commercial DEIPA alone.

Among the DEG-based mixtures, CH and NEW contents remained consistently lower than those of the TIPA- and DEIPA-based systems, particularly at early ages. Unlike alkanolamines, DEG lacks an amine functional group and therefore does not promote hydration through ion-complexation mechanisms with clinker phases. Nevertheless, modification with propanoic acid substantially enhanced the development of hydration. At 28 days, the M-DEG-BFS1-45 mixture exhibited a mass loss of 3.164% in the 50–200 °C range, together with CH and NEW contents of 30.086% and 15.224%, respectively, compared with 2.896%, 26.437%, and 13.028% for the corresponding DEG-BFS1-45 mixture. This improvement is consistent with the enhanced grinding performance of M-DEG, which produced a finer cement with lower D_50_ and D_90_ values and a narrower particle-size distribution, thereby facilitating hydration.

The 28-day CH and NEW contents of the P-DEG-BFS1-45 mixture were slightly higher than those of the commercial DEG mixture but remained lower than those of M-DEG-BFS1-45. These findings indicate that PCE hybridization provided only a limited enhancement in the DEG-based system, whereas modification with propanoic acid produced a substantially greater improvement. This behavior demonstrates that a given modification strategy does not produce the same response across TIPA-, DEIPA-, and DEG-based additives, and that its effectiveness is strongly dependent on the chemical structure of the original GA.

Overall, the TGA-derived parameters of the BFS1-containing mixtures were lower than those of the corresponding mixtures without BFS1. This reduction primarily reflects the decrease in portlandite-producing clinker resulting from replacing 45% of the binder with BFS1. Nevertheless, the progressive increase in low-temperature mass loss, CH content, and NEW with curing time indicates that hydration continued throughout the 28 days. The latent hydraulic reaction of BFS1, promoted by its predominantly glassy structure and relatively high Al_2_O_3_ and MgO contents, contributed to the formation of secondary hydration products, including C-(A)-S-H, AFm, and hydrotalcite-like phases at later ages. Importantly, the contribution of hydrotalcite-like phases to the low-temperature mass loss cannot be quantitatively separated using the present three-interval TGA approach. Consequently, the low-temperature mass loss and NEW values should be regarded as comparative parameters that reflect the combined contribution of several thermally labile hydration products rather than as phase-specific quantities. However, the lower CH contents observed in the BFS1-containing mixtures should not be attributed solely to portlandite consumption during slag hydration. Instead, they reflect the combined effects of clinker dilution and the progressive hydration of BFS1.

Among the BFS1-containing mixtures, M-TIPA exhibited the greatest degree of hydration development, as evidenced by the highest 28-day low-temperature mass loss, CH content, and NEW content. M-DEIPA and M-DEG likewise produced higher CH and NEW values than their corresponding commercial additives. Although the magnitude of improvement varied by grinding aid type, organic acid modification consistently enhanced the hydration-promoting effect of the commercial additives, particularly at later hydration ages. These comparisons are considered robust at a relative level; however, the absolute contributions of individual hydration phases to the TGA-derived values remain uncertain due to overlapping thermal decomposition events.

#### 3.4.2. BFS2 Paste Mixtures

Table 8 presents the TGA results at 24 h, 3 days, and 28 days for pastes containing 45% by mass BFS2 as a substitute, produced using control cement and cements containing 0.05% GA.

In mixtures containing BFS2, all GAs increased the mass loss in the 50–200 °C range compared with the additive-free C-BFS2-45 mixture. At 24 h, the mass loss increased from 2.322% for the control mixture to 2.749%, 2.770%, and 2.704% for the TIPA-, M-TIPA-, and P-TIPA-containing mixtures, respectively. The corresponding CH contents were 13.354%, 14.947%, and 12.305%, all substantially higher than the 7.503% measured for the control mixture. The higher low-temperature mass loss, together with the higher CH contents, suggests enhanced early-age hydration in the TIPA-containing systems. However, the 50–200 °C mass loss includes overlapping contributions from several hydration products and cannot be assigned exclusively to newly formed C-(A)-S-H or other individual phases. In particular, water associated with AFm-type and Mg-Al hydrotalcite-like phases may contribute to this temperature interval in BFS-containing systems.

Among the TIPA-based mixtures, M-TIPA exhibited the greatest hydration-promoting effect. The mass loss in the 50–200 °C range increased to 3.573% at 3 days and 4.318% at 28 days. At 28 days, the CH and NEW contents reached 36.945% and 18.934%, respectively, representing the highest values among all BFS2-containing mixtures. Compared with commercial TIPA, hexanoic acid modification consistently produced higher low-temperature mass loss, CH content, and NEW content, indicating enhanced hydration development. This improvement can be attributed to the combined effects of the finer particle-size distribution produced during grinding and the modified interaction between M-TIPA and clinker phases during hydration [42,63].

In contrast, the P-TIPA-BFS2-45 mixture consistently exhibited lower CH and NEW contents than both the TIPA- and M-TIPA-containing mixtures. This behavior is likely associated with two concurrent effects. First, adsorption of PCE molecules onto cement particle surfaces, along with the steric hindrance generated by their side chains, can delay ion dissolution, nucleation, and early hydrate precipitation. Second, because the total GA dosage was kept constant in the hybrid systems, the actual amount of TIPA was reduced to half that used in the corresponding single-component formulation. The combined influence of these factors accounts for the comparatively lower degree of hydration observed in the P-TIPA system [46,47,53].

A similar trend was observed within the DEIPA-based mixtures. At 24 h, the mass losses in the 50–200 °C range were 2.742%, 2.799%, and 2.365% for the DEIPA-, M-DEIPA-, and P-DEIPA-containing mixtures, respectively. Although P-DEIPA produced only a limited increase in low-temperature mass loss, both DEIPA and particularly M-DEIPA promoted greater hydrate formation during the early hydration period. Throughout the curing period, the M-DEIPA-BFS2-45 mixture consistently exhibited higher CH and NEW contents than both the commercial DEIPA and P-DEIPA mixtures. At 28 days, its low-temperature mass loss, CH content, and NEW content reached 4.054%, 32.869%, and 16.982%, respectively. These results indicate that organic acid modification effectively enhanced the hydration-promoting ability of DEIPA, whereas hybridization with PCE provided only a limited improvement. Nevertheless, the magnitudes of individual phase contributions to the low-temperature mass loss cannot be resolved with the present TGA intervals.

The DEG-based mixtures exhibited lower CH contents at early ages than the corresponding amine-based systems, reflecting the absence of the amine functionality responsible for the ion-complexation mechanisms that promote clinker hydration in TIPA- and DEIPA-based additives. At 24 h, the mass losses in the 50–200 °C range were 2.625%, 2.733%, and 2.600% for the DEG-, M-DEG-, and P-DEG-containing mixtures, respectively. Nevertheless, propanoic acid modification substantially improved DEG’s hydration performance. In the M-DEG-BFS2-45 mixture, the 28-day low-temperature mass loss increased to 3.984%, while the CH and NEW contents reached 30.816% and 15.972%, respectively. These improvements suggest that propanoic acid modification enhanced the grinding performance of DEG, resulting in a finer cement with a more favorable particle size distribution and, consequently, greater hydration product formation in the BFS2-containing system.

In all mixtures containing BFS2, both mass losses in the 50–200 °C range and NEW values increased with age. This trend is consistent with continued hydration and hydrate formation; however, it should not be interpreted as direct evidence of a specific hydration phase, as the thermal decomposition ranges overlap. The finer particle size distribution and higher CaO/SiO_2_ ratio of BFS2 increased the surface area exposed to the alkaline pore solution and enhanced the tendency of the glassy phase to dissolve. However, the total CaO content within the BFS was not considered simply as free or directly “reactive CaO”; instead, slag reactivity was evaluated based on the combined effects of the CaO/SiO_2_ ratio, glassy phase structure, Al_2_O_3_ and MgO contents, and particle fineness [8,10,15].

When comparing the results for BFS1 and BFS2, the mass loss in the 50–200 °C range for the additive-free BFS2 mixture was higher than that of the C-BFS1-45 mixture at all ages. This difference is consistent with the finer particle size distribution and higher basicity of BFS2. However, in mixtures containing GA, the relative performance of the two slags varied depending on the type of additive. While BFS2 increased low-temperature mass loss at later ages—particularly in systems containing DEIPA, M-DEIPA, DEG, M-DEG, and P-DEG—it yielded lower values than BFS1 in mixtures based on TIPA and P-TIPA. This behavior demonstrates that slag properties and GA chemistry are not independent and that the same additive does not produce identical hydration development across different slags. In both BFS groups, M-TIPA yielded the highest 28-day CH and NEW values. While M-DEIPA and M-DEG also achieved higher values than their commercial counterparts, PCE-hybridized admixtures generally underperformed compared to the organic acid-modified admixtures. Overall, the TGA results support the comparative interpretation that organic acid modification promoted hydration-related development more consistently than PCE hybridization. However, because the contributions of C-(A)-S-H, AFm-type phases, and Mg-Al hydrotalcite-like phases cannot be quantitatively separated within the selected temperature intervals, the TGA-derived low-temperature mass loss and NEW values are interpreted as comparative indicators rather than phase-specific or absolute measures of hydration.

### 3.5. SEM Analysis Results of the Mixtures

**Figure 3:**
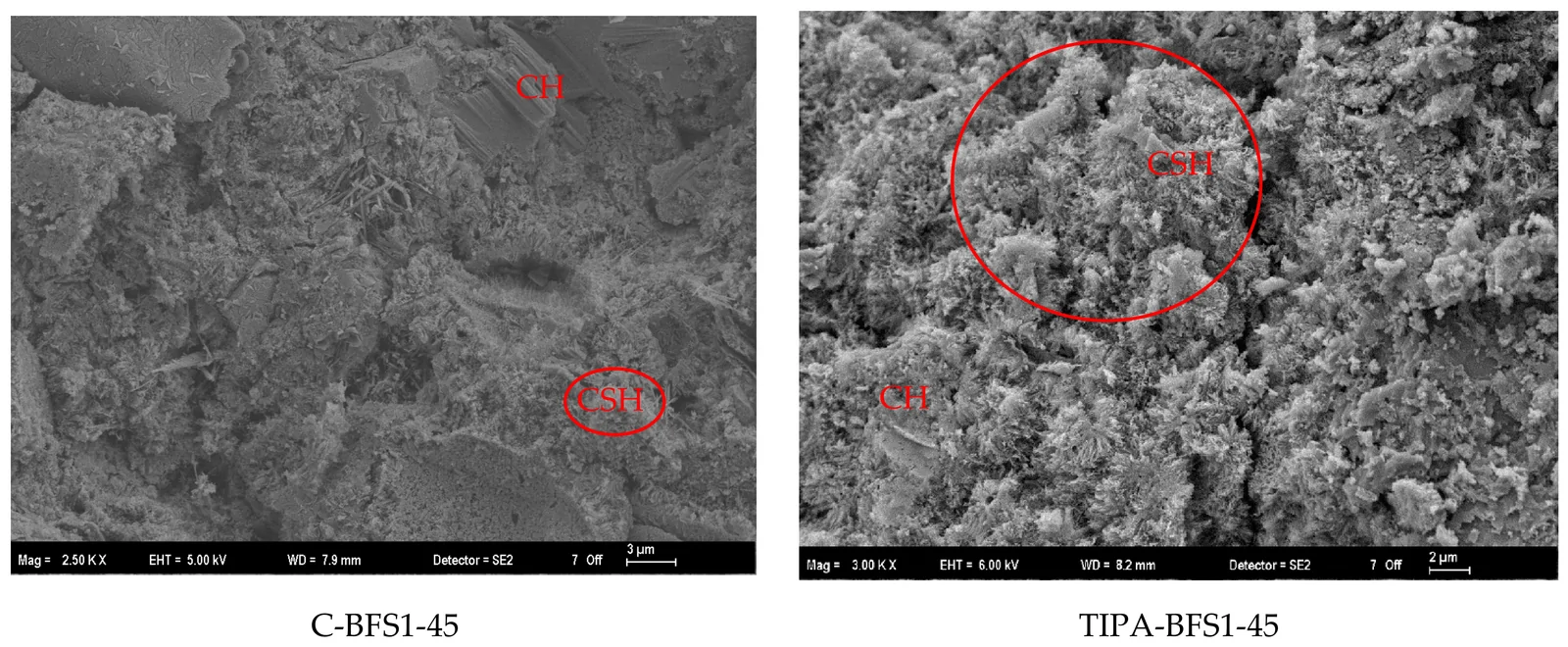

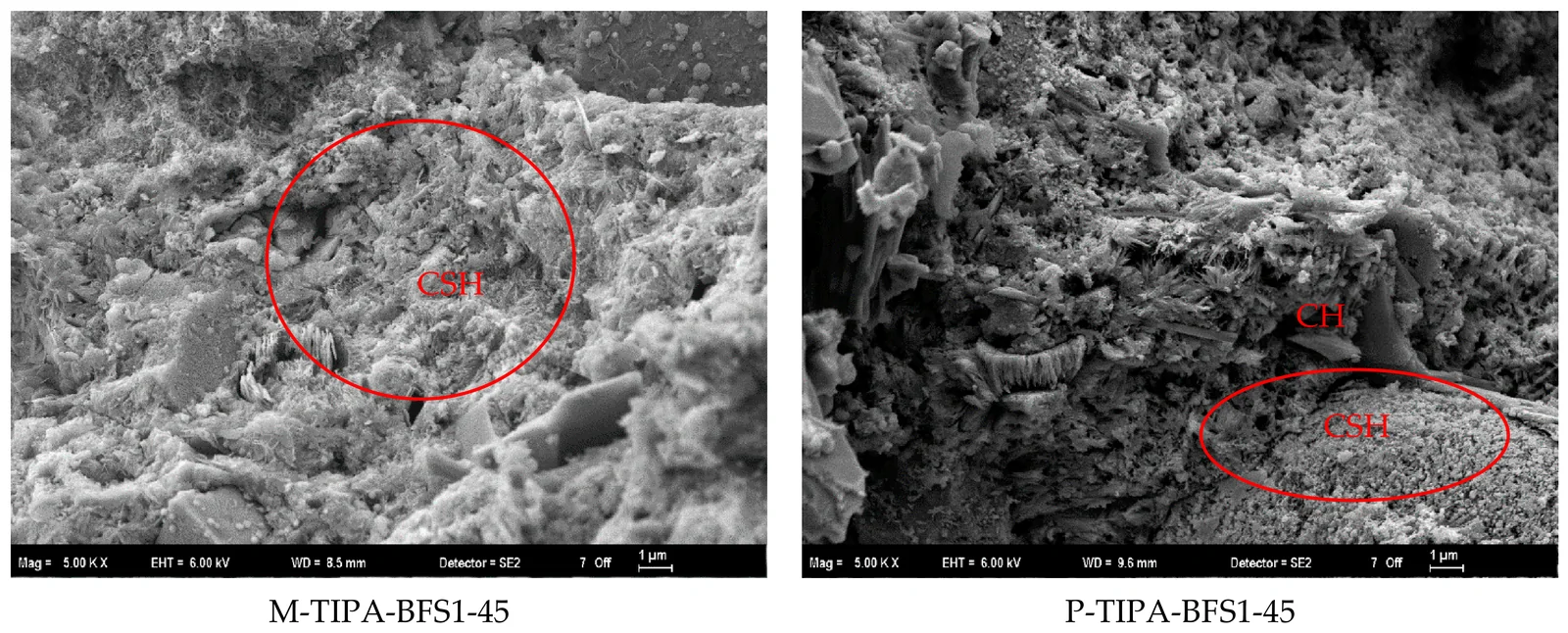
28-day SEM images of C, TIPA, M-TIPA, and P-TIPA pastes with 45% BFS1 substitution.

**Figure 4:**
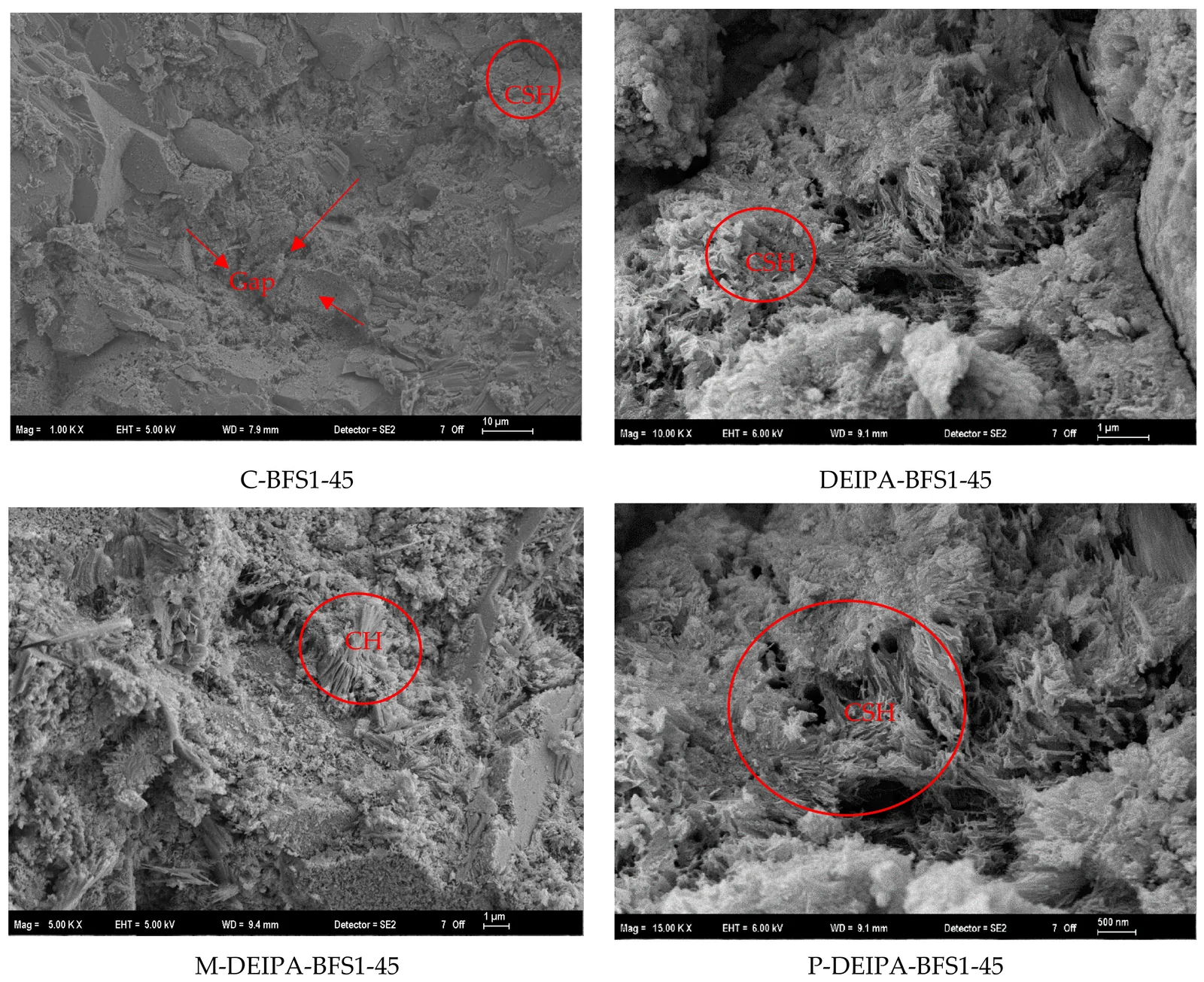
28-day SEM images of C, DEIPA, M-DEIPA, and P-DEIPA pastes with 45% BFS1 substitution.

**Figure 5:**
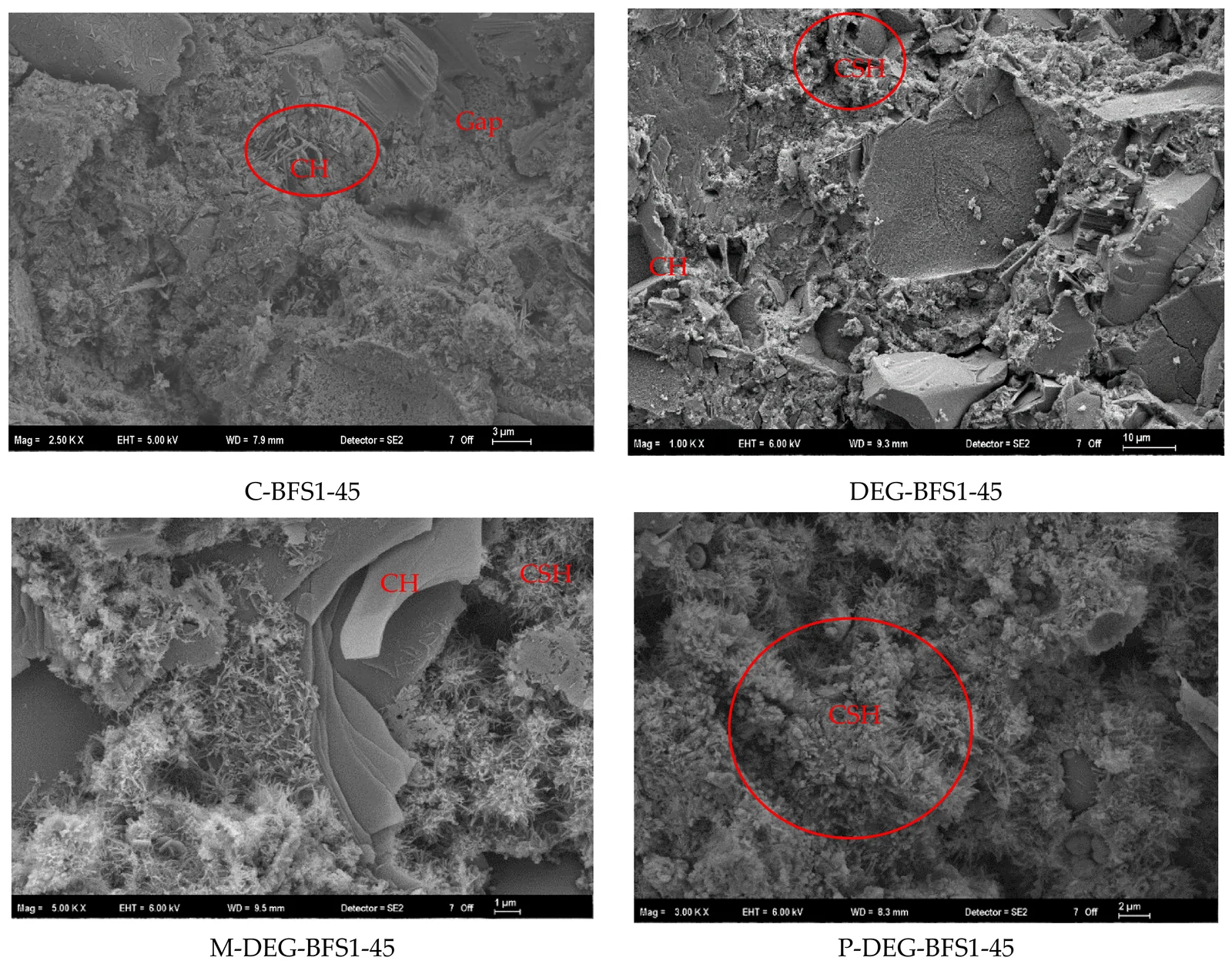
28-day SEM images of C, DEG, M-DEG, and P-DEG pastes with 45% BFS1 substitution.

Figure 3, Figure 4 and Figure 5 present the 28-day SEM images of pastes containing 45% BFS1 replacement by mass, prepared using control cement and cements with 0.05% GA. Since SEM images reflect the local morphology of selected fracture surfaces, the evaluations were based on the form, distribution, and relationships between hydration products and particle surfaces, rather than on direct measurements of porosity.

In the C-BFS1-45 paste, irregular coarse particles, plate-like crystals, and fine hydration products are observed simultaneously surrounding these features. The presence of partially uncovered grain surfaces and occasional open pores within the matrix suggests that some clinker or slag particles remained unreacted or only partially reacted after 28 days of hydration with 45% BFS1 replacement. The observed gel-like products exhibit a morphology consistent with C-(A)-S-H phases, whereas the plate-like crystals may correspond to portlandite or AFm-type hydrates. However, in the absence of EDS analysis, these phase assignments should be considered morphological interpretations supported by XRD and TGA results rather than definitive phase identifications [9,65,68].

Compared with the control paste, the TIPA-BFS1-45 paste exhibited a higher abundance of fine, fibrous, and gel-like hydration products that developed around coarse particles. The more extensive coverage of particle surfaces by hydration products is consistent with TIPA’s ability to promote clinker hydration. This behavior also aligns with the reduction in D_50_ and, in particular, D_90_ values observed upon TIPA addition, indicating a decrease in the coarse-particle fraction and an increase in the available surface area for hydration. Previous studies have shown that grinding aids modify the particle size distribution of cement by preventing the agglomeration of fine particles during grinding [27,69].

The M-TIPA-BFS1-45 paste exhibited a more developed hydration morphology, characterized by widely distributed fine fibrous, lamellar, and gel-like products. In addition, the regions between larger particles were extensively filled with fine hydration products. This microstructural development is consistent with the highest 28-day CH and NEW values obtained for M-TIPA among all BFS1-containing mixtures. Notably, the M-TIPA-containing cement showed a more advanced hydration morphology despite exhibiting higher D_50_ and D_90_ values than the commercial TIPA mixture. This indicates that the improved hydration performance of M-TIPA cannot be attributed solely to enhanced grinding efficiency or particle refinement. The higher hydration development of M-TIPA may be associated with a modified interaction of the additive with clinker surfaces and dissolved ions after hexanoic acid modification, as suggested by previous studies [42,44].

In the P-TIPA-BFS1-45 paste, gel-like products and plate-like crystals were observed together with partially reacted coarse particles. However, the distribution and coverage of hydration products on particle surfaces appeared less uniform than in the M-TIPA-BFS1-45 paste. This observation is consistent with the lower 28-day CH and NEW values obtained for P-TIPA-BFS1-45 compared with M-TIPA-BFS1-45. Although PCE hybridization influenced the particle-size distribution, the hydration enhancement remained below that achieved with M-TIPA. This is consistent with the reduced proportion of TIPA in the hybrid additive; an additional influence of the PCE component on surface reactions cannot be excluded but was not directly verified.

In the DEIPA-BFS1-45 and P-DEIPA-BFS1-45 pastes, fibrous and gel-like hydration products are observed developing around voids and coarse particles. Although fine hydration products are widely distributed throughout both matrices, some localized open regions remain, indicating that the pore structure has not been filled after 28 days. In contrast, the M-DEIPA-BFS1-45 paste exhibits a more interconnected hydration morphology, in which plate-like and fibrous products form a denser network, and a more distinct hydrate layer develops around coarse particles. This microstructural feature is consistent with the higher 28-day CH and NEW values obtained for M-DEIPA-BFS1-45 compared with the commercial DEIPA and P-DEIPA mixtures. These results indicate that organic acid modification improves the hydration-promoting effect of DEIPA, whereas hybridization with PCE does not provide a comparable enhancement.

In the DEG-BFS1-45 paste, partially reacted coarse particles coexist with fine hydration products formed around their surfaces. The M-DEG-BFS1-45 paste exhibits a more developed morphology, characterized by distinct plate-like crystals, fibrous products, and gel-like regions. Meanwhile, the P-DEG-BFS1-45 paste exhibits a finer, more widely distributed hydration structure. The higher 28-day CH and NEW values for the M-DEG-BFS1-45 mixture than for the DEG-BFS1-45 and P-DEG-BFS1-45 mixtures indicate that propanoic acid modification enhances DEG’s hydration-promoting capability.

Overall, the SEM observations of BFS1-containing mixtures indicated differences in the development and spatial distribution of hydration products after 28 days, depending on the GA’s chemical structure and modification method. However, the observed morphological features were not considered definitive phase identification, as the SEM analysis was based solely on secondary-electron imaging without complementary EDS analysis. Among the organic acid-modified additives, M-TIPA exhibited the most pronounced microstructural development, characterized by a more extensive distribution of hydration products and higher TGA-derived hydration parameters. Similarly, M-DEIPA and M-DEG produced more developed hydration structures compared with their corresponding commercial additives. However, the influence of PCE hybridization varied with the chemical structure of the original GA, leading to different effects on hydration product formation across the TIPA-, DEIPA-, and DEG-based systems.

**Figure 6:**
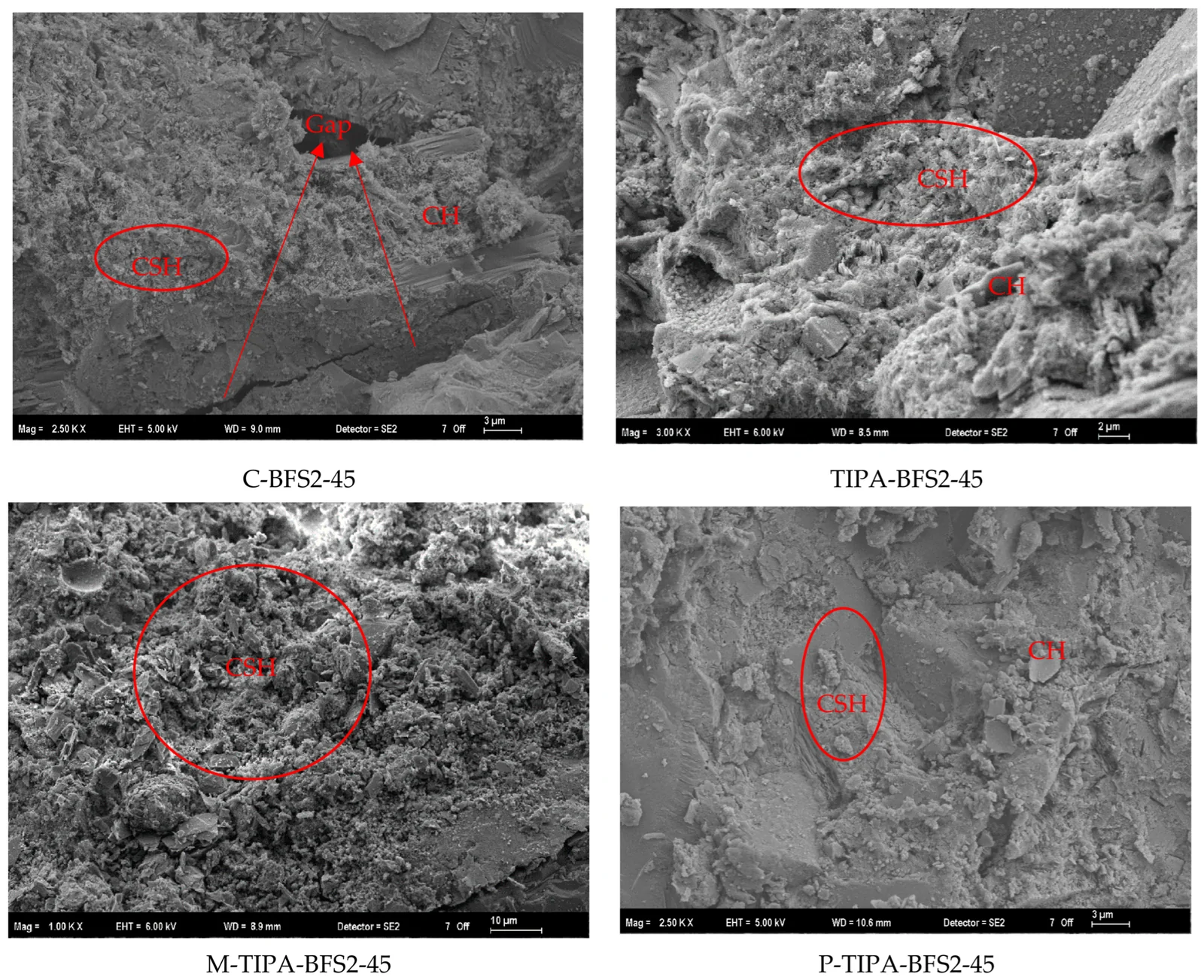
28-day SEM images of C, TIPA, M-TIPA, and P-TIPA pastes with 45% BFS2 substitution.

**Figure 7:**
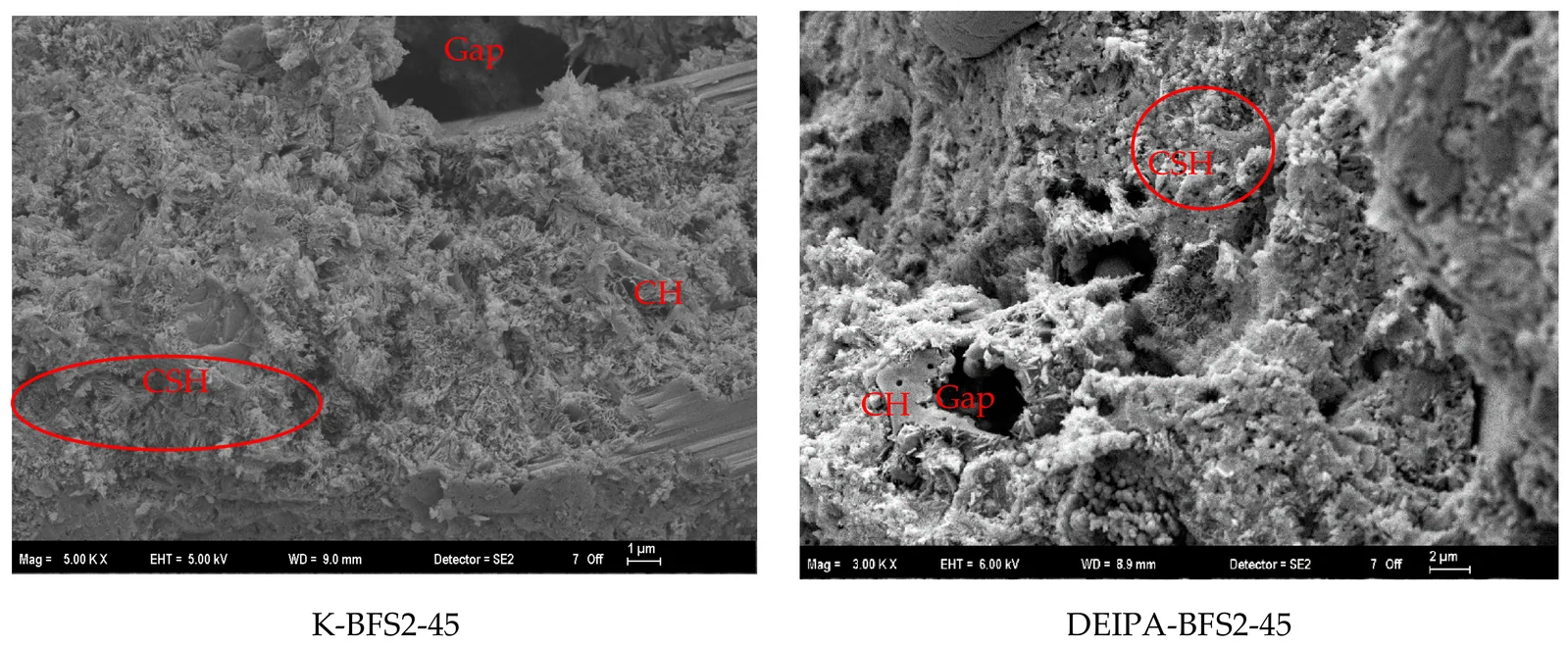

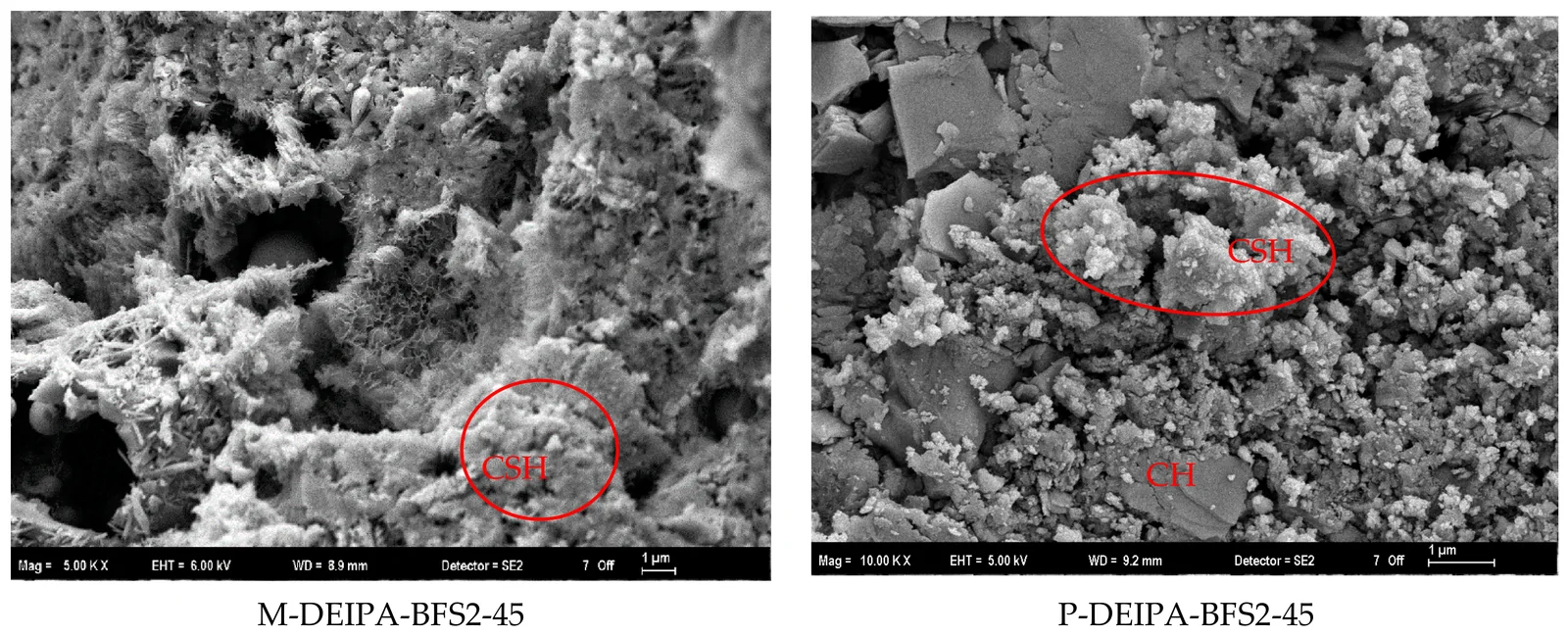
28-day SEM images of C, DEIPA, M-DEIPA, and P-DEIPA pastes with 45% BFS2 substitution.

**Figure 8:**
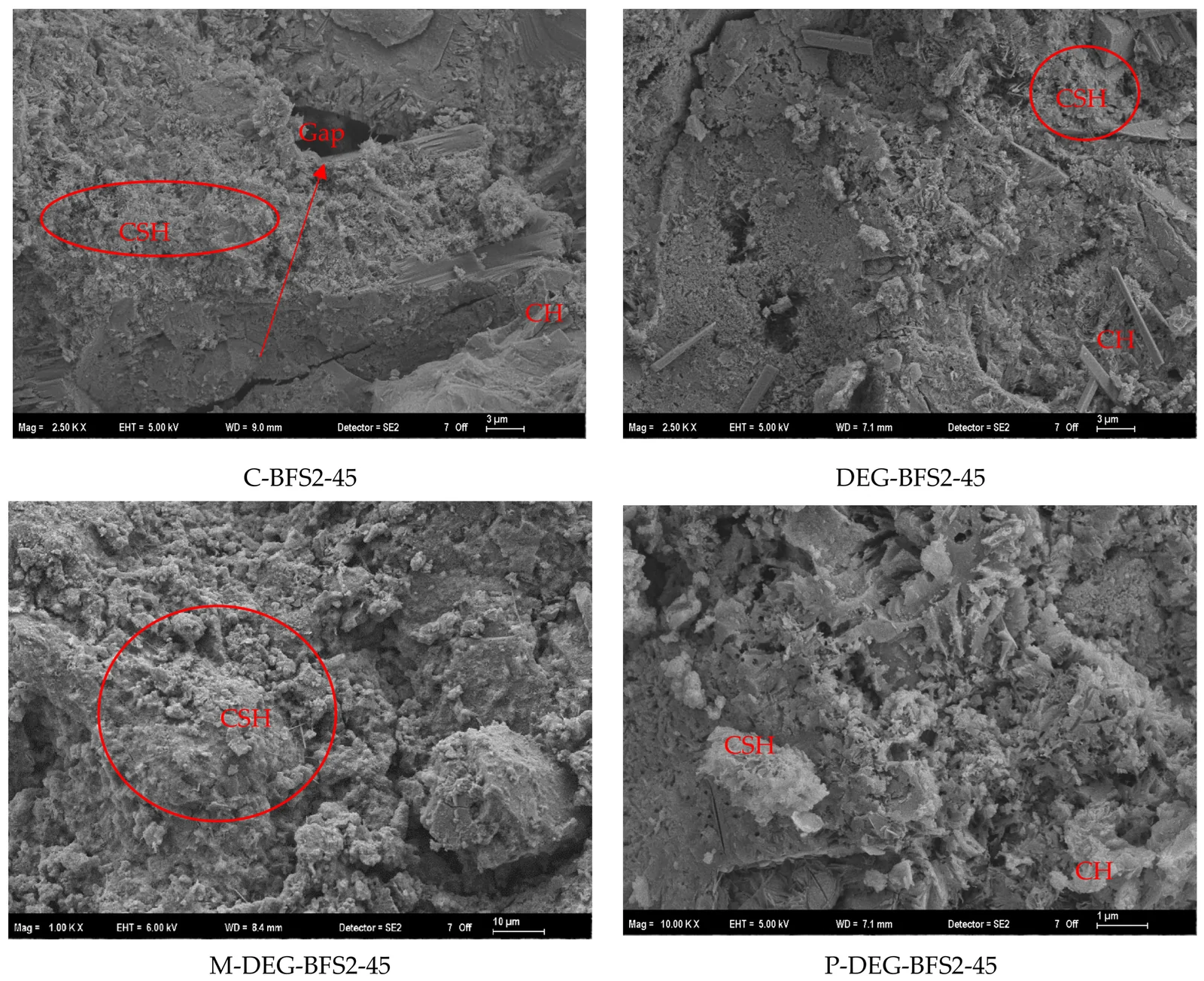
28-day SEM images of C, DEG, M-DEG, and P-DEG pastes with 45% BFS2 substitution.

Figure 6, Figure 7 and Figure 8 present the 28-day SEM images of pastes containing 45% BFS2 replacement by mass, including the control cement and cements produced with 0.05% GA.

In the C-BFS2-45 paste, gel-like hydration products are observed around coarse grains, although open voids and discontinuous regions remain visible. These features indicate the presence of partially reacted particles after 28 days, as expected for systems with a 45% BFS replacement. Compared with BFS1, the finer particle size distribution and higher CaO/SiO_2_ ratio of BFS2 provide a larger reactive surface area and promote dissolution of the glassy phase, contributing to enhanced hydration development [7,9].

Among the TIPA-based mixtures, M-TIPA-BFS2-45 exhibited a more developed morphology, characterized by widely distributed fibrous, needle-like, and gel-like hydration products. This observation agrees with its highest 28-day CH and NEW values among the BFS2-containing mixtures. In contrast, P-TIPA-BFS2-45 showed partially reacted coarse particles accompanied by fine hydration products, and its lower TGA-derived parameters indicate that PCE hybridization provided less improvement than organic acid modification.

The DEIPA- and DEG-based systems showed similar trends. M-DEIPA-BFS2-45 exhibited more developed hydration-product layers around coarse particles and in the vicinity of voids compared with commercial DEIPA and P-DEIPA, consistent with its higher CH and NEW values. Likewise, M-DEG-BFS2-45 exhibited a more extensive distribution of hydration-product features than DEG-BFS2-45 and P-DEG-BFS2-45, supporting the beneficial effect of propanoic acid modification in DEG-based systems. As with the BFS1-containing mixtures, these SEM observations represent qualitative morphological evidence and should not be interpreted as definitive identification of individual hydration phases or as quantitative characterization of the pore structure.

**Figure 9:**
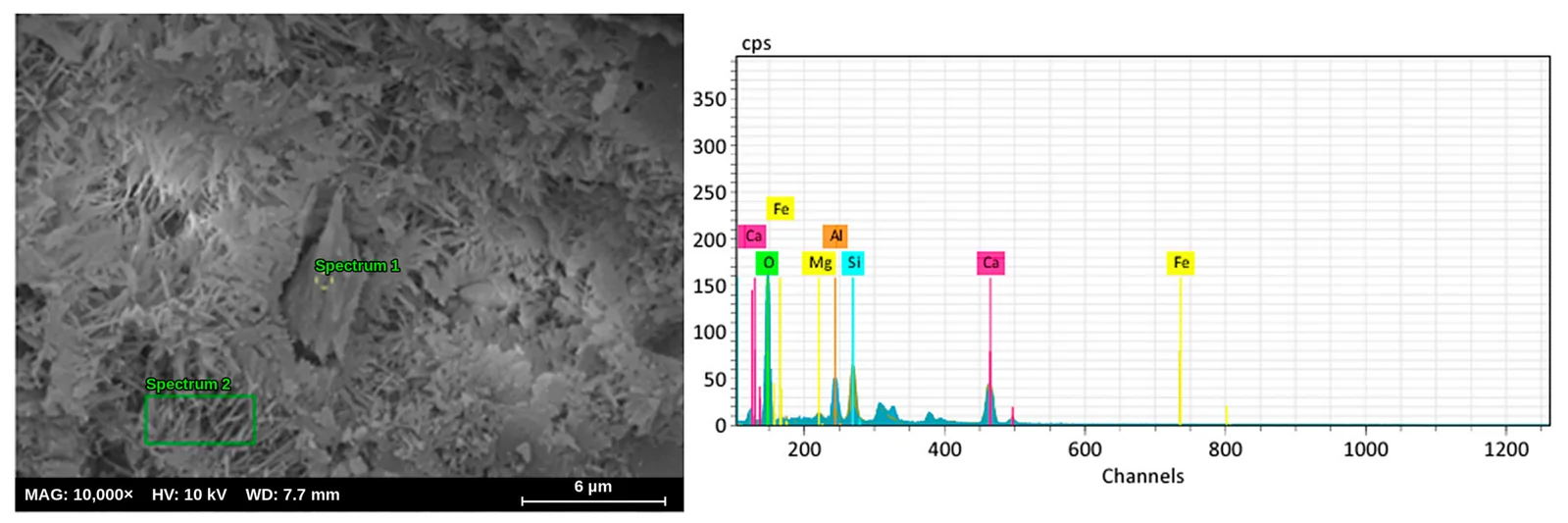
SEM-EDS images of the control mixture (without GA and BFS).

**Figure 10:**
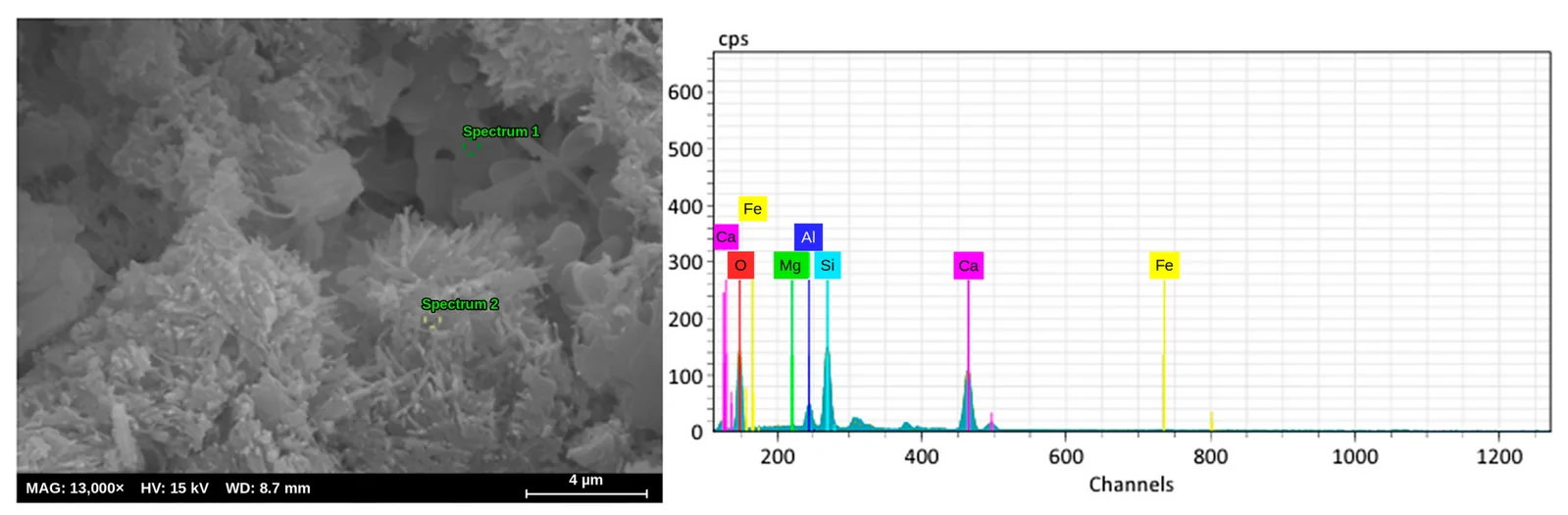
SEM-EDS images of the 45% BFS1 substituted control mixture.

Considering BFS1 and BFS2 systems together, SEM observations revealed gel-like C-(A)-S-H products, plate-like crystals, and partially reacted grains in all mixtures. The greater presence of fine hydration products in some BFS2-containing mixtures is consistent with the finer particle-size distribution and the distinct chemical composition of BFS2. Although its higher nominal basicity may contribute to glass dissolution, the SEM observations do not allow the individual effects of particle fineness, glass structure, CaO/SiO_2_ ratio, and Al_2_O_3_/MgO contents to be distinguished. However, because SEM images represent localized regions, direct comparisons of overall porosity or matrix densification between BFS1 and BFS2 could not be made. The combined evaluation of SEM, XRD, and TGA results demonstrates that organic acid modification, particularly M-TIPA, effectively promotes the development of hydration products in BFS-containing systems. M-DEIPA and M-DEG also generated more developed microstructures and higher CH and NEW values than their commercial counterparts. The influence of PCE hybridization depended on the chemical structure of the original GA and the characteristics of the BFS used. To complement the morphological observations with compositional data, SEM-EDS images and normalized elemental mass concentrations are presented for the control paste without GA in Figure 9 and Table 9, and for the control paste with 45% BFS1 replacement in Figure 10 and Table 10. Because the effects of the GA are discussed in the preceding sections based on bulk TGA and mechanical results, these two pastes were selected to illustrate the effect of slag replacement on the composition of the hydration products, not the effect of the GA.

In the BFS-free paste, spots on fibrous and foil-like products showed Ca/Si ratios of 1.09–1.89 and Al/Si ratios of 0.15–0.26. A third spot (Ca/Si = 3.41; Ca ≈ 62 mass %) was located in a Ca-rich region, which is consistent with calcium hydroxide or calcium carbonate intermixed with a gel-like product. In the paste containing 45% BFS1, the two spots on gel-like products gave Ca/Si ratios of 0.65–0.67 and Al/Si ratios of 0.32, i.e., lower Ca/Si and higher Al/Si than in the BFS-free paste. This trend is qualitatively consistent with the compositions in Table 3: the clinker is strongly Ca-rich (molar CaO/SiO_2_ ≈ 5.0), whereas BFS1 has a molar Ca/Si of 0.88 and an Al/Si of 0.43. The incorporation of species released from the Al-rich slag glass is expected to lower the Ca/Si ratio and raise the Al/Si ratio of the gel. This agrees with the earlier discussion of Al incorporation into C-(A)-S-H and AFm-type phases for BFS1 (Section 3.2) [9,65,67]. No Ca-rich spot was found among the analyzed spots of the BFS-containing paste, which is in line with the lower portlandite contents determined by TGA and XRD for the BFS pastes. These lower contents were attributed mainly to clinker dilution at early ages and to progressively increasing slag reaction at later ages (Section 3.3 and Section 3.4). Owing to the small number of analyzed spots and the finite interaction volume of EDS, which may include intermixed phases, these data are considered indicative of trends rather than of quantitative gel compositions.

### 3.6. Compressive Strength of Mixtures

The 1-, 3-, 7-, 28-, and 56-day compressive strengths of mortars prepared with control cement (without grinding aids), commercial grinding aids (TIPA, DEIPA, and DEG), organic acid-modified grinding aids (M-TIPA, M-DEIPA, and M-DEG), and PCE-hybridized grinding aids (P-TIPA, P-DEIPA, and P-DEG) were determined. The strength development was evaluated by considering the combined effects of grinding aid type and BFS replacement level, with particular emphasis on differences in early- and later-age strength performance.

#### 3.6.1. BFS1 Mortars

The compressive strength results of mortars with and without BFS1 are presented in Table 11.

Increasing the BFS1 replacement ratio reduced compressive strength across all GA groups. For the control mortar (K) without GA, the 28-day compressive strength decreased from 43.20 MPa at 0% BFS1 replacement to 37.21, 31.20, and 19.98 MPa at 30%, 45%, and 60% replacement levels, corresponding to reductions of 13.9%, 27.8%, and 53.8%, respectively. The corresponding 56-day strengths were 42.03, 35.82, and 21.84 MPa, indicating that the increasing replacement ratio intensified the dilution effect by reducing the amount of clinker available for strength development.

The relatively low Blaine fineness of BFS1 (3200 cm^2^/g) and its coarse particle-size distribution (D_50_ = 27.2 µm and D_90_ = 112 µm), compared with ground cement, likely limited its early contribution to strength development. The reduced strength loss observed at 30% replacement at later ages indicates that the latent hydraulic reaction of BFS1 gradually contributed to strength development. However, at higher replacement levels, particularly 45% and 60%, the contribution of slag hydration was insufficient to compensate for the reduced clinker content fully. Previous studies have similarly shown that the strength development of slag-containing cements depends on several factors, including slag replacement ratio, curing age, glassy phase structure, and fineness [70,71].

In the absence of BFS1, the influence of GA type on compressive strength varied with hydration age. Although DEG exhibited the highest 1-day strength (17.50 MPa), the highest 28- and 56-day strengths were achieved with M-TIPA, at 53.20 and 58.52 MPa, respectively. While the 1-day strength of the TIPA-containing mortar was lower than that of the control mortar, its 28- and 56-day strengths exceeded the control by 10.9% and 12.8%, respectively. This indicates that TIPA’s contribution becomes more significant at intermediate and later ages. Ref. Sun et. al. [72] suggested that the influence of TIPA on strength development may be associated not only with hydration degree but also with improvements in the cement paste–aggregate interface.

Within the TIPA-based systems, M-TIPA exhibited the highest strength values at all BFS1 replacement levels. The M-TIPA-BFS1-30 mortar achieved 28- and 56-day strengths of 45.82 and 52.69 MPa, respectively, corresponding to increases of 6.1% and 12.8% compared with the BFS-free control mortar. For the M-TIPA-BFS1-45 and M-TIPA-BFS1-60 mixtures, the corresponding strengths were 38.42/44.85 MPa and 24.60/27.40 MPa at 28/56 days, respectively. Overall, M-TIPA increased 28- and 56-day strengths by approximately 11% compared with commercial TIPA at equivalent BFS1 replacement levels.

P-TIPA also improved strength development compared with commercial TIPA but remained inferior to M-TIPA. At 30% BFS1 replacement, the P-TIPA mixture reached 42.11 MPa at 28 days and 48.43 MPa at 56 days. Although PCE hybridization enhanced early-age strength by improving particle dispersion, the effect of organic acid modification became more evident at intermediate and later ages. This suggests that PCE dispersion effects and TIPA-induced hydration enhancement act synergistically; however, because the total admixture dosage was held constant, the reduced proportion of TIPA in the hybrid system limited the long-term strength improvement.

In the DEIPA-based systems, M-DEIPA consistently produced higher later-age strengths than both commercial DEIPA and P-DEIPA. For the M-DEIPA-BFS1-30 mortar, the 28- and 56-day strengths were 43.45 and 50.01 MPa, respectively, representing increases of 8.3% and 8.4% over the corresponding commercial DEIPA mixture. A similar trend was observed at 45% and 60% BFS1 replacement levels. Although P-DEIPA provided a notable improvement in early strength, reaching 31.68 MPa at 7 days for the 30% BFS1 replacement mixture and exceeding the corresponding DEIPA and M-DEIPA mixtures, M-DEIPA exhibited superior performance at later ages.

The influence of alkanolamines on strength development extends beyond accelerating early hydration; they also affect the dissolution–precipitation processes of C_3_A, C_4_AF, and silicate phases, the composition of hydration products, and the cement paste–aggregate interface. Their effectiveness strongly depends on the activator type and dosage [73].

Although DEG provided relatively high 1-day strengths, this advantage was not maintained at later ages. For DEG-BFS1-30, the 28- and 56-day strengths were 38.87 and 44.71 MPa, respectively, increasing to 40.29 and 46.37 MPa with M-DEG, corresponding to an improvement of approximately 3.7%. In contrast, P-DEG showed lower strengths (37.37 and 43.45 MPa) than commercial DEG. Thus, PCE hybridization appears less effective in DEG-based systems than in TIPA- and DEIPA-based systems. Considering all BFS1-containing mixtures, M-TIPA exhibited the highest 28- and 56-day compressive strengths at all replacement levels (30%, 45%, and 60%). M-DEIPA showed the second-best performance, whereas P-DEIPA and P-TIPA produced comparable strength values. Although increasing BFS1 replacement to 60% resulted in a substantial strength reduction, the M-TIPA-BFS1-60 mortar achieved 23.1% and 25.5% higher strengths than the additive-free C-BFS1-60 mixture at 28 and 56 days, respectively. This demonstrates that GA modification can partially mitigate the strength loss associated with high BFS replacement levels by enhancing hydration development; however, it cannot fully offset the reduction in clinker content due to dilution.

#### 3.6.2. BFS2 Mortars

The compressive strength results of mortars with and without BFS2 are presented in Table 12.

BFS2 replacement reduced compressive strength at all ages, with the reduction becoming more pronounced as the replacement level increased. The 28-day strengths of the C-BFS2-30, C-BFS2-45, and C-BFS2-60 mortars were 38.21, 30.31, and 18.60 MPa, respectively, corresponding to reductions of 11.6%, 29.8%, and 56.9% compared with the BFS-free control mortar (K). Their 56-day strengths increased to 43.88, 35.68, and 23.30 MPa, respectively. Particularly, the 25.3% strength gain between 28 and 56 days for C-BFS2-60 indicates that the delayed hydraulic contribution of BFS2 becomes more significant at high replacement levels.

BFS2 exhibited a Blaine fineness of 3500 cm^2^/g, with D_50_ and D_90_ values of 22.2 and 87.5 µm, respectively. Although BFS2 was finer and had a higher CaO/SiO_2_ ratio than BFS1, its particle size remained coarser than that of ground cement. Therefore, its latent hydraulic contribution was insufficient to compensate for clinker dilution, particularly at early ages. Strength development in BFS-containing systems depends on slag composition, fineness, and replacement level, while CaO content alone cannot be considered a direct indicator of slag reactivity [70,71].

Among the TIPA-based mixtures, TIPA-BFS2-30 achieved 28- and 56-day strengths of 42.02 and 48.92 MPa, respectively, approaching and exceeding the strength of the BFS-free control mortar. However, at 45% and 60% BFS2 replacement, TIPA improved strength relative to the additive-free mixtures but did not fully compensate for the dilution effect. In contrast, M-TIPA provided the highest strength values among all BFS2-containing mixtures. The M-TIPA-BFS2-30 mortar reached 46.55 MPa at 28 days and 54.13 MPa at 56 days, representing increases of 7.8% and 15.9% compared with the BFS-free control mortar and approximately 10.8% and 10.7% compared with commercial TIPA at the same replacement level. At 45% and 60% BFS2 replacement, M-TIPA produced strengths of 36.82–43.93 MPa and 22.53–28.55 MPa at 28 and 56 days, respectively.

The M-TIPA-BFS2-60 mortar exhibited a 26.7% increase in strength between 28 and 56 days, whereas the corresponding BFS1 mixture increased by only 11.4%. This confirms the higher long-term reactivity of BFS2 at high replacement levels, which is associated with its finer particle size and higher basicity. However, BFS2 did not provide higher 28-day strengths than BFS1 at 45% and 60% replacement levels, indicating that the relative contribution of the two slags depends on both curing age and replacement ratio.

For the DEIPA-based systems, the 28- and 56-day strengths of the DEIPA-BFS2-30 mortar increased from 41.14 and 47.92 MPa to 44.25 and 51.45 MPa with M-DEIPA, corresponding to an improvement of approximately 7.5%. Although P-DEIPA provided the highest 7-day strength within the DEIPA group (31.72 MPa), M-DEIPA exhibited superior performance at later ages. The same trend was observed for the 45% and 60% BFS2 replacement levels.

The influence of TIPA- and DEIPA-based admixtures on strength development arises from the combined effects of changes in grinding-induced particle-size distribution and ionic interactions during hydration. Alkanolamines can interact with Fe- and Al-containing phases, modifying the hydration kinetics of intermediate and silicate phases. Previous studies have shown that ligand-type admixtures influence the onset of reactions and the development of strength in slag-containing systems. However, their effectiveness depends on binder chemistry and activator conditions [73]. Similarly, the influence of TIPA and other alkanolamines is governed by admixture dosage, clinker mineralogy, and the type of supplementary binder used [63,72].

For DEG-BFS2-30, the 28- and 56-day strengths increased from 39.86 and 46.42 MPa with commercial DEG to 41.35 and 48.14 MPa with M-DEG, corresponding to an improvement of approximately 3.7% at later ages. Although P-DEG provided relatively high early-age strength, its 28- and 56-day performance remained below that of DEG and M-DEG, indicating that PCE hybridization mainly improved early particle dispersion but provided limited benefits for later-age strength development.

Considering both BFS1 and BFS2 systems, M-TIPA consistently achieved the highest 28- and 56-day strengths at all replacement levels, followed by M-DEIPA. P-DEIPA and P-TIPA improved strength compared with their commercial counterparts but remained below the organic acid-modified systems. M-DEG provided only limited improvement over commercial DEG, whereas P-DEG showed lower performance at later ages.

At 30% BFS replacement, M-TIPA-containing mortars exceeded the strength of the BFS-free control mortar at both 28 and 56 days. At 45% replacement, modified alkanolamines substantially reduced strength losses but could not fully compensate for clinker dilution. At 60% replacement, clinker dilution became the dominant factor; however, M-TIPA and M-DEIPA still provided higher strengths than the corresponding BFS mixtures without admixtures. Overall, organic acid modification enhanced the medium- and long-term performance of TIPA- and DEIPA-based GAs. In contrast, the effect of PCE hybridization depended strongly on the chemical structure of the base alkanolamine.

## 4. Conclusions

This study investigated commercial TIPA, DEIPA, and DEG; their organic acid-modified derivatives; and PCE-hybridized formulations in binder systems containing two BFS types. The results obtained are summarized below:

The differences observed among cements with similar Blaine fineness values confirmed that grinding performance cannot be evaluated solely by specific surface area. TIPA was more effective at reducing coarse particles, whereas M-DEG produced a narrower particle-size distribution. The effect of PCE hybridization depended on the base GA chemistry: P-TIPA and P-DEIPA improved particle distribution, whereas P-DEG was less effective at reducing the coarse fraction. Thus, modification strategies do not produce identical effects across different GA chemistries.

Organic acid modification provided a stronger enhancement of hydration development than commercial and PCE-hybridized additives. M-TIPA showed the most pronounced improvement in portlandite development, TGA-based hydration indicators, and microstructural evolution, while M-DEIPA and M-DEG also outperformed their commercial counterparts. PCE-hybridized additives yielded lower hydration indicators than the organic-acid-modified additives. This is attributed primarily to the halved alkanolamine/glycol content of the 1:1 hybrid formulations; a contribution of PCE adsorption, or of competitive adsorption between the hybrid components, is possible but was not directly verified in this study. BFS replacement reduced Portlandite formation mainly due to early-age clinker dilution, while slag reactions increasingly influenced phase development at later ages.

Differences between BFS1 and BFS2 were associated with both chemical composition and particle characteristics. The finer BFS2, with its higher CaO/SiO_2_ ratio, promoted earlier dissolution and hydration, whereas the higher Al_2_O_3_ and MgO contents of BFS1 affected later-age hydration products. Therefore, slag reactivity depends not only on CaO content or basicity but also on fineness, the composition of glassy phases, and GA compatibility.

Increasing BFS replacement generally reduced compressive strength, although the extent depended strongly on GA type. At 30% replacement, M-TIPA-containing mortars exceeded the BFS-free control for both slag types, reaching 45.82/52.69 MPa for BFS1 and 46.55/54.13 MPa for BFS2 at 28/56 days. M-DEIPA was the second most effective modified GA, while P-TIPA and P-DEIPA provided moderate improvements. At higher replacement levels, modified alkanolamines mitigated strength losses but could not fully overcome clinker dilution. Nevertheless, M-TIPA consistently provided the highest 28- and 56-day strengths for both BFS types, while BFS2 showed better long-term performance, particularly at higher replacement levels.

Overall, the results demonstrate that organic acid modification influences GA performance beyond grinding, affecting subsequent hydration and strength development. M-TIPA exhibited the most pronounced overall effect through the combined contribution of improved particle size distribution and modified hydration behavior. The comparison between organic acid modification and PCE hybridization highlights the importance of base-additive chemistry and modification strategies when designing grinding aids for BFS-blended cement. From a polymer-related perspective, the findings further indicate that PCE-containing hybrid formulations should be evaluated in conjunction with their effects on particle characteristics and the interfacial properties of ground cement, rather than on dispersion or fineness indicators alone. The findings indicate that GA selection for BFS-based binders should consider not only additive type but also molecular structure, modification strategy, slag fineness, chemical composition, and replacement level.

Future studies could establish a cross-scale explanatory chain by coupling molecular-level simulations of interfacial interactions between organic acid-modified grinding aids and clinker and slag surfaces with the microstructural evolution of hydration products and the macroscopic compressive strength of mortars, following the molecular-to-macroscopic research paradigm used in other material systems. Combining such simulations with direct surface characterization techniques and quantitative adsorption measurements would help clarify the interactions of modified grinding aids and PCE-containing formulations with clinker and slag surfaces.

## Figures and Tables

**Table 1:** Major studies on cement-based systems containing GA and BFS.

Source	The System Under Investigation	Key Findings
[25]	Various commercial GAs	The effects of different types of additives on grindability, fineness, and cement performance vary.
[32]	TIPA, DEIPA, DEG and EG	Amine and glycol-based additives have produced different results on grinding, particle size distribution, rheology, and strength.
[59]	Portland cement containing DEIPA and EDIPA	While low dosages promote aluminate, ferrite, and silicate reactions, high dosages have limited hydration and strength.
[18]	A binder containing high-volume BFS and steel slag	Increased fineness has improved early hydration and the development of the microstructure.
[35]	Portland cement containing alkanolamine	Alkanolamines have altered phase dissolution and early hydrates
[36]	Model cements containing TIPA and DEIPA	The effects of TIPA and DEIPA on the silicate, aluminate, and ferrite phases are not the same.
[43]	TEA Modified with an Ester	Modification altered the grinding quality and particle size distribution.
[41]	Modified TEA	The TEA modification has both increased grinding efficiency and improved the properties of the cement-based system.
[24]	Various GA types	Different particle size distributions and rheological behaviors were observed at similar fineness levels.
[53]	PCE type GAs with different pH	PCE performance is sensitive to changes in pH and dosage.
[56]	PCE-based GA and a binder containing fly ash	The pH and dosage of the admixtures altered the sequence of hydration and strength development.
[57]	BFS, ground with TEA	A low TEA dosage increased slag reaction and strength, whereas a high dosage delayed them.
[58]	50% BFS and various TEA concentrations	A low dosage promoted early reactions, while a high dosage suppressed silicate hydration.
[40]	TIPA, DEIPA, and DEG combined with PCE	Hybrid additives altered agglomeration and powder flow; the effects varied by additive type.
[42,44]	PCE-based GAs with modified and different molecular structures	Molecular structure collectively influenced grinding efficiency, particle size distribution, hydration, and rheology.

**Table 2:** (**a**) Some Properties of Organic Acids Used in Commercial GAs and Modification. (**b**) Some molecular properties of a PCE-based GA.

(**a**)					
**Chemical**	**Solid Content (%)**	**Molecular Formula**	**Molar Mass (g/mol)**	**Density (g/cm^3^)**	**pH. 25 °C**
TIPA	85	C_9_H_21_NO_3_	191.27	1.12	10.8
DEIPA	85	C_7_H_17_NO_3_	163.21	1.08	9.8
DEG	99	C_4_H_10_O_3_	106.12	1.11	7.2
Hexanoic acid	99	CH_3_(CH_2_)_4_COOH	116.16	0.93	-
Propionic acid	99	CH_3_CH_2_COOH	74.08	-	-
(**b**)					
**Properties**			**Value**		
Main chain length (g/mol)			4000		
Side chain length (g/mol)			2400		
Weight-average molecular weight (g/mol)			37.000		
Side chain/main chain ratio			14 *		

* The value “14” does not represent the ratio of 2400 to 4000. This parameter is a molecular structure indicator calculated based on the average number of side chains attached to a main chain and the relative molecular weights of the chains.

**Table 3:** Chemical compositions and certain physical properties of clinker, gypsum, and BFS.

Oxide/Component or Property	Clinker	Gypsum	BFS1	BFS2
SiO_2_ (%)	15.43	2.63	38.91	34.45
Al_2_O_3_ (%)	3.15	1.11	14.24	11.81
Fe_2_O_3_ (%)	4.46	0.68	0.95	1.34
CaO (%)	72.02	41.98	32.09	40.52
MgO (%)	0.29	0.35	7.68	5.03
SO_3_ (%)	0.78	52.75	1.53	1.84
Na_2_O (%)	0.39	0.07	0.45	0.43
K2O (%)	0.96	0.14	1.12	0.86
Others (%)	1.98	0.29	2.71	3.58
Cl^−^ (%)	0.013	0.000	0.010	0.013
Loss of ignition (%)	0.52	—	0.31	0.12
C_3_S (%)	58.81	—	—	—
C_2_S (%)	21.76	—	—	—
C_3_A (%)	8.79	—	—	—
C_4_AF (%)	10.64	—	—	—
Specific Gravity	—	—	2.27	2.31
Blaine specific surface area (cm^2^/g)	—	—	4200	4300

**Table 4:** Blaine specific surface areas and particle size distribution parameters of cements and BFSs.

Cements and Pozzolanas	Blaine (cm^2^/g)	0–10 μm	10–32 μm	32–45 μm	45–60 μm	60–90 μm	D50	D90	n
Control	3810	37.56	30.72	12.43	10.57	8.72	17.3	69.9	0.804
TIPA	3910	39.17	37.49	12.2	7.19	3.95	14.9	49.2	0.931
M-TIPA	3880	39.51	36.41	13.09	7.07	3.92	16.5	55.2	0.929
P-TIPA	3900	37.23	35.05	13.81	8.18	5.73	16.7	56.1	0.918
DEIPA	3940	38.52	34.62	12.32	8.08	6.46	16.3	64.6	0.863
M-DEIPA	3840	39.15	33.5	12.64	7.81	6.9	16.5	68.2	0.964
P-DEIPA	3920	37.83	32.66	14.26	9.04	6.21	15.1	53.9	0.942
DEG	3900	35.74	33.85	13.67	9.78	6.96	16.5	56.3	0.966
M-DEG	3880	37.42	33.84	13.23	9.28	6.23	15.2	54.7	0.998
P-DEG	3910	39.23	34.46	11.47	8.00	6.84	16.7	77.7	0.978
BFS1	3200	30.9	32.63	12.94	10.83	12.93	27.2	112	0.814
BFS2	3500	30.77	35.37	14.14	10.37	10.38	22.2	87.5	0.837

**Table 5:** Portlandite peak intensities of paste containing and not containing BFS1; comparative indicator only.

Mixtures	24-h	3-Day	28-Day
C	20.43	44.64	95.13
C-BFS1-45	16.13	30.36	51.96
TIPA	38.26	78.12	163.08
TIPA-BFS1-45	30.00	54.12	88.33
M-TIPA	49.29	96.72	199.32
M-TIPA-BFS1-45	37.50	67.32	109.12
P-TIPA	34.96	70.68	144.96
P-TIPA-BFS1-45	27.00	48.84	77.94
DEIPA	26.55	53.94	117.78
DEIPA-BFS1-45	21.00	38.28	62.35
M-DEIPA	40.14	79.98	172.14
M-DEIPA-BFS1-45	30.75	55.44	90.93
P-DEIPA	25.97	52.08	113.25
P-DEIPA-BFS1-45	20.25	36.96	59.75
DEG	21.22	42.78	95.13
DEG-BFS1-45	16.88	31.02	51.96
M-DEG	31.86	63.24	135.90
M-DEG-BFS1-45	24.75	44.88	72.74
P-DEG	18.47	37.20	81.54
P-DEG-BFS1-45	14.63	27.06	45.43

**Table 6:** Portlandite peak intensities of pastes containing and not containing BFS2; comparative indicator only.

Mixture	24-h	3-Day	28-Day
C	20.43	44.64	95.13
C-BFS2-45	16.88	32.34	49.36
TIPA	38.26	78.12	163.08
TIPA-BFS2-45	30.75	55.44	80.54
M-TIPA	49.29	96.72	199.32
M-TIPA-BFS2-45	38.25	68.64	93.53
P-TIPA	34.96	70.68	144.96
P-TIPA-BFS2-45	27.75	50.16	75.34
DEIPA	26.55	53.94	117.78
DEIPA-BFS2-45	21.75	39.60	57.16
M-DEIPA	40.14	79.98	172.14
M-DEIPA-BFS2-45	31.50	56.76	77.94
P-DEIPA	25.97	52.08	113.25
P-DEIPA-BFS2-45	21.00	38.28	57.16
DEG	21.22	42.78	95.13
DEG-BFS2-45	17.25	31.68	48.06
M-DEG	31.86	63.24	135.90
M-DEG-BFS2-45	25.50	46.20	67.55
P-DEG	18.47	37.20	81.54
P-DEG-BFS2-45	15.00	27.72	44.17

**Table 7:** TGA results of pastes containing 45% BFS1 by mass at 24 h, 3 days, and 28 days.

Mixtures	24-h					3-Day					28-Day				
	Weight Loss (%)			CH Content	NEW (%)	Weight Loss (%)			CH Content	NEW (%)	Weight Loss (%)			CH Content	NEW (%)
	50–200 °C	200–390 °C	400–550 °C			50–200 °C	200–390 °C	400–550 °C			50–200 °C	200–390 °C	400–550 °C		
C	2.033	0.678	1.491	12.428	9.482	2.643	0.949	2.386	23.570	12.431	3.105	1.042	2.864	28.917	14.200
C-BFS1-45	1.26	0.434	0.984	9.310	7.103	1.563	0.590	1.456	17.436	9.196	1.823	0.742	1.965	21.284	11.732
TIPA	3.977	1.088	3.165	19.704	10.378	5.17	1.523	5.064	41.432	19.179	5.851	1.638	6.982	52.147	23.418
TIPA-BFS1-45	2.466	0.696	2.089	13.897	7.319	3.058	0.947	3.092	28.001	12.962	3.218	1.146	3.876	34.915	17.684
M-TIPA	4.229	1.172	3.344	21.899	11.275	5.498	1.641	5.35	47.428	21.107	6.307	1.718	7.483	56.463	25.047
M-TIPA-BFS1-45	2.707	0.727	2.207	15.851	8.161	4.006	0.988	2.737	30.095	13.393	4.456	1.182	4.214	37.862	19.438
P-TIPA	4.261	1.149	3.389	19.910	10.587	5.539	1.609	5.422	35.380	13.911	5.102	1.569	6.137	45.674	20.126
P-TIPA-BFS1-45	2.727	0.712	2.237	13.809	7.343	4.036	0.969	2.774	22.521	8.855	4.284	1.128	4.098	29.476	14.982
DEIPA	4.116	1.372	2.896	19.026	10.506	5.351	1.921	4.634	34.715	13.868	5.197	1.694	6.089	46.283	20.648
DEIPA-BFS1-45	2.634	0.851	1.911	13.451	7.428	3.899	1.157	2.37	21.773	8.698	3.147	1.264	3.942	28.905	14.216
M-DEIPA	3.638	1.188	2.747	18.906	11.781	4.729	1.663	4.395	35.579	16.258	5.497	1.748	6.436	48.963	22.347
M-DEIPA-BFS1-45	2.401	0.737	1.703	13.118	8.175	2.977	1.09	2.521	23.822	10.886	2.964	1.182	3.468	30.214	15.106
P-DEIPA	4.219	1.379	3.297	19.934	10.397	5.485	1.931	5.275	36.998	14.972	5.612	1.726	6.334	47.564	21.538
P-DEIPA-BFS1-45	2.785	0.883	2.11	13.709	7.150	3.787	1.456	2.616	22.333	9.038	3.238	1.382	3.986	29.118	14.684
DEG	3.56	1.294	2.481	16.785	10.324	4.628	1.812	3.97	30.875	13.690	4.852	1.621	5.436	42.086	19.138
DEG-BFS1-45	2.35	0.828	1.588	11.869	7.300	3.195	1.226	1.969	19.844	8.799	2.896	1.104	3.214	26.437	13.028
M-DEG	4.019	1.159	2.937	19.182	10.413	5.225	1.623	4.699	35.524	14.245	5.302	1.579	5.986	45.387	20.436
M-DEG-BFS1-45	2.653	0.742	1.88	13.386	7.267	3.607	1.009	2.782	24.110	9.668	3.164	1.198	3.642	30.086	15.224
P-DEG	3.824	1.355	2.594	17.184	10.371	4.971	1.897	4.15	31.340	13.627	5.051	1.628	5.583	43.164	19.673
P-DEG-BFS1-45	2.524	0.867	1.608	11.813	7.129	3.145	1.179	2.38	21.073	9.163	2.984	1.176	3.318	26.915	13.412

**Table 8:** TGA results of pastes containing 45% BFS2 by mass at 24 h, 3 days, and 28 days.

Mixtures	24-h					3-Day					28-Day				
	Weight Loss (%)			CH Content	NEW (%)	Weight Loss (%)			CH Content	NEW (%)	Weight Loss (%)			CH Content	NEW (%)
	50–200 °C	200–390 °C	400–550 °C			50–200 °C	200–390 °C	400–550 °C			50–200 °C	200–390 °C	400–550 °C		
C	2.033	0.678	1.491	12.428	9.482	2.643	0.949	2.386	23.570	12.431	3.105	1.042	2.864	28.917	14.2
C-BFS2-45	2.322	0.74	1.375	7.503	5.724	2.74	0.893	2.115	18.501	9.757	2.985	0.922	2.421	24.873	12.118
TIPA	3.977	1.088	3.165	19.704	10.378	5.17	1.523	5.064	41.432	19.179	5.851	1.638	6.982	52.147	23.418
TIPA-BFS2-45	2.749	0.785	2.207	13.354	7.034	3.546	1.052	3.443	27.799	12.868	3.892	1.204	4.214	33.684	16.972
M-TIPA	4.229	1.172	3.344	21.899	11.275	5.498	1.641	5.35	47.428	21.107	6.307	1.718	7.483	56.463	25.047
M-TIPA-BFS2-45	2.77	0.77	2.237	14.947	7.696	3.573	1.032	3.489	31.542	14.037	4.318	1.286	4.736	36.945	18.934
P-TIPA	4.261	1.149	3.389	19.910	10.587	5.539	1.609	5.422	35.380	13.911	5.102	1.569	6.137	45.674	20.126
P-TIPA-BFS2-45	2.704	0.707	2.121	12.305	6.543	3.462	0.948	3.223	22.723	8.934	3.647	1.173	3.984	28.916	14.885
DEIPA	4.116	1.372	2.896	19.026	10.506	5.351	1.921	4.634	34.715	13.868	5.197	1.694	6.089	46.283	20.648
DEIPA-BFS2-45	2.742	0.892	1.911	12.424	6.860	3.611	1.195	2.982	22.644	9.046	3.721	1.338	3.846	29.274	15.104
M-DEIPA	3.638	1.188	2.747	18.906	11.781	4.729	1.663	4.395	35.579	16.258	5.497	1.748	6.436	48.963	22.347
M-DEIPA-BFS2-45	2.799	0.91	2.176	14.035	8.746	3.51	1.201	3.351	25.590	11.693	4.054	1.421	4.417	32.869	16.982
P-DEIPA	4.219	1.379	3.297	19.934	10.397	5.485	1.931	5.275	36.998	14.972	5.612	1.726	6.334	47.564	21.538
P-DEIPA-BFS2-45	2.365	0.796	1.813	11.460	5.977	3.05	1.051	2.828	21.560	8.725	3.412	1.262	3.538	27.148	13.967
DEG	3.56	1.294	2.481	16.785	10.324	4.628	1.812	3.97	30.875	13.690	4.852	1.621	5.436	42.086	19.138
DEG-BFS2-45	2.625	0.841	1.738	11.458	7.047	2.962	1.11	2.669	21.077	9.346	3.506	1.287	3.425	27.694	14.318
M-DEG	4.019	1.159	2.937	19.182	10.413	5.225	1.623	4.699	35.524	14.245	5.302	1.579	5.986	45.387	20.436
M-DEG-BFS2-45	2.733	0.777	1.88	12.359	6.709	3.498	1.025	2.895	22.930	9.195	3.984	1.349	4.006	30.816	15.972
P-DEG	3.824	1.355	2.594	17.184	10.371	4.971	1.897	4.15	31.340	13.627	5.051	1.628	5.583	43.164	19.673
P-DEG-BFS2-45	2.6	0.881	1.708	11.196	6.757	3.328	1.163	2.567	20.197	8.782	3.318	1.305	3.291	26.487	13.704

**Table 9:** Normalized mass concentration of control mixture (%).

Spectrum	Oxygen	Magnesium	Aluminium	Silicon	Calcium	Iron
Spectrum 1	59.75	0.71	10.1	4.14	24.42	0.88
Spectrum 2	48.02	0.32	2.27	17.18	30.05	2.16

**Table 10:** Normalized mass concentration of 45% BFS1 substituted control mixture (%).

Spectrum	Oxygen	Magnesium	Aluminium	Silicon	Calcium	Iron
Spectrum 1	50.63	0.45	6.17	20.11	19.15	3.49
Spectrum 2	53.77	0.3	5.94	19.47	18.14	2.38

**Table 11:** The compressive strength results of mortars with and without BFS1.

Mixture	1-Day (MPa)	3-Day (MPa)	7-Day (MPa)	28-Day (MPa)	56-Day (MPa)
C	14.40	29.01	38.30	43.20	46.70
C-BFS1-30	10.59	22.44	30.89	37.21	42.03
C-BFS1-45	8.13	17.55	24.64	31.20	35.82
C-BFS1-60	4.93	11.22	16.02	19.98	21.84
TIPA	12.30	28.20	35.81	47.90	52.69
TIPA-BFS1-30	9.01	21.80	28.84	41.24	47.48
TIPA-BFS1-45	6.96	17.03	23.01	34.58	40.43
TIPA-BFS1-60	4.22	10.91	14.96	22.13	24.64
M-TIPA	12.80	28.71	39.73	53.20	58.52
M-TIPA-BFS1-30	9.40	22.20	32.00	45.82	52.69
M-TIPA-BFS1-45	7.22	17.36	25.55	38.42	44.85
M-TIPA-BFS1-60	4.40	11.13	16.59	24.60	27.40
P-TIPA	13.90	28.70	37.30	48.90	53.79
P-TIPA-BFS1-30	10.19	22.20	30.02	42.11	48.43
P-TIPA-BFS1-45	7.87	17.36	23.99	35.30	41.28
P-TIPA-BFS1-60	4.75	11.13	15.58	22.57	25.16
DEIPA	14.70	26.40	35.30	46.60	51.26
DEIPA-BFS1-30	10.82	20.46	28.44	40.13	46.14
DEIPA-BFS1-45	8.32	15.99	22.75	33.61	39.33
DEIPA-BFS1-60	5.06	10.21	14.74	21.52	24.00
M-DEIPA	15.20	30.20	37.17	50.50	55.55
M-DEIPA-BFS1-30	11.14	23.38	29.94	43.45	50.01
M-DEIPA-BFS1-45	8.58	18.27	23.92	36.47	42.58
M-DEIPA-BFS1-60	5.24	11.70	15.53	23.32	26.00
P-DEIPA	14.20	29.40	39.30	49.26	54.19
P-DEIPA-BFS1-30	10.43	22.75	31.68	42.42	48.82
P-DEIPA-BFS1-45	8.06	17.75	25.29	35.56	41.60
P-DEIPA-BFS1-60	4.88	11.40	16.41	22.75	25.36
DEG	17.50	28.78	34.10	45.10	49.61
DEG-BFS1-30	12.88	22.28	27.49	38.87	44.71
DEG-BFS1-45	9.88	17.42	21.97	32.57	38.03
DEG-BFS1-60	6.03	11.13	14.26	20.86	23.20
M-DEG	13.20	26.50	33.80	46.80	51.48
M-DEG-BFS1-30	9.72	20.54	27.26	40.29	46.37
M-DEG-BFS1-45	7.48	15.99	21.78	33.80	39.46
M-DEG-BFS1-60	4.53	10.25	14.12	21.60	24.08
P-DEG	14.90	30.10	38.60	43.40	48.26
P-DEG-BFS1-30	10.98	23.31	31.13	37.37	43.45
P-DEG-BFS1-45	8.45	18.20	24.83	31.33	36.99
P-DEG-BFS1-60	5.10	11.66	16.15	20.06	22.56

**Table 12:** The compressive strength results of mortars with and without BFS2.

Mixture	1-Day (MPa)	3-Day (MPa)	7-Day (MPa)	28-Day (MPa)	56-Day (MPa)
C	14.40	29.01	38.30	43.20	46.70
C-BFS2-30	10.37	21.98	30.95	38.21	43.88
C-BFS2-45	7.45	16.19	23.78	30.31	35.68
C-BFS2-60	4.18	9.24	14.21	18.60	23.30
TIPA	12.30	28.20	35.81	47.90	52.69
TIPA-BFS2-30	8.86	21.23	28.92	42.02	48.92
TIPA-BFS2-45	6.39	15.78	22.36	33.48	39.93
TIPA-BFS2-60	3.61	9.00	13.39	20.45	25.98
M-TIPA	12.80	28.71	39.73	53.20	58.52
M-TIPA-BFS2-30	9.18	21.74	32.02	46.55	54.13
M-TIPA-BFS2-45	6.65	16.01	24.61	36.82	43.93
M-TIPA-BFS2-60	3.75	9.18	14.75	22.53	28.55
P-TIPA	13.90	28.70	37.30	48.90	53.79
P-TIPA-BFS2-30	9.97	21.70	30.06	42.88	49.93
P-TIPA-BFS2-45	7.24	16.07	23.28	34.12	40.75
P-TIPA-BFS2-60	4.03	9.13	13.85	20.77	26.36
DEIPA	14.70	26.40	35.30	46.60	51.26
DEIPA-BFS2-30	10.60	20.03	28.64	41.14	47.92
DEIPA-BFS2-45	7.64	14.77	22.10	32.66	39.01
DEIPA-BFS2-60	4.30	8.47	13.25	20.01	25.42
M-DEIPA	15.20	30.20	37.17	50.50	55.55
M-DEIPA-BFS2-30	10.92	22.79	29.97	44.25	51.45
M-DEIPA-BFS2-45	7.90	16.88	23.17	35.12	41.90
M-DEIPA-BFS2-60	4.44	9.68	13.91	21.54	27.30
P-DEIPA	14.20	29.40	39.30	49.26	54.19
P-DEIPA-BFS2-30	10.21	22.25	31.72	43.31	50.38
P-DEIPA-BFS2-45	7.44	16.52	24.54	34.51	41.15
P-DEIPA-BFS2-60	4.16	9.39	14.62	21.03	26.67
DEG	17.50	28.78	34.10	45.10	49.61
DEG-BFS2-30	12.66	21.85	27.66	39.86	46.42
DEG-BFS2-45	9.09	16.09	21.31	31.57	37.73
DEG-BFS2-60	5.13	9.23	12.85	19.44	24.71
M-DEG	13.20	26.50	33.80	46.80	51.48
M-DEG-BFS2-30	9.50	20.10	27.45	41.35	48.14
M-DEG-BFS2-45	6.85	14.84	21.20	32.79	39.14
M-DEG-BFS2-60	3.85	8.45	12.70	20.03	25.46
P-DEG	14.90	30.10	38.60	43.40	48.26
P-DEG-BFS2-30	10.76	22.85	31.24	38.52	45.31
P-DEG-BFS2-45	7.77	16.90	24.07	30.57	36.92
P-DEG-BFS2-60	4.35	9.59	14.32	18.69	24.01

## Data Availability

All data generated or analyzed during this study are included in this published article.
